# A unified approach for sparse dynamical system inference from temporal measurements

**DOI:** 10.1093/bioinformatics/btz065

**Published:** 2018-01-31

**Authors:** Yannis Pantazis, Ioannis Tsamardinos

**Affiliations:** 1 Institute of Applied and Computational Mathematics, Foundation for Research and Technology - Hellas (FORTH), Heraklion, Greece; 2 Department of Computer Science, University of Crete, Heraklion, Greece; 3 Gnosis Data Analysis PC, Heraklion, Greece

## Abstract

**Motivation:**

Temporal variations in biological systems and more generally in natural sciences are typically modeled as a set of ordinary, partial or stochastic differential or difference equations. Algorithms for learning the structure and the parameters of a dynamical system are distinguished based on whether time is discrete or continuous, observations are time-series or time-course and whether the system is deterministic or stochastic, however, there is no approach able to handle the various types of dynamical systems simultaneously.

**Results:**

In this paper, we present a unified approach to infer both the structure and the parameters of non-linear dynamical systems of any type under the restriction of being linear with respect to the unknown parameters. Our approach, which is named Unified Sparse Dynamics Learning (USDL), constitutes of two steps. First, an atemporal system of equations is derived through the application of the weak formulation. Then, assuming a sparse representation for the dynamical system, we show that the inference problem can be expressed as a sparse signal recovery problem, allowing the application of an extensive body of algorithms and theoretical results. Results on simulated data demonstrate the efficacy and superiority of the USDL algorithm under multiple interventions and/or stochasticity. Additionally, USDL’s accuracy significantly correlates with theoretical metrics such as the exact recovery coefficient. On real single-cell data, the proposed approach is able to induce high-confidence subgraphs of the signaling pathway.

**Availability and implementation:**

Source code is available at *Bioinformatics* online. USDL algorithm has been also integrated in SCENERY (http://scenery.csd.uoc.gr/); an online tool for single-cell mass cytometry analytics.

**Supplementary information:**

Supplementary data are available at *Bioinformatics* online.

## 1 Introduction

The great majority of biological processes are time-varying requiring the use of dynamical models for their quantitative description. Examples range from macroscopic processes studied for instance in epidemiology and population dynamics to microscopic processes such as biochemical reactions and gene regulation in a living cell, all of which are modeled as time-varying dynamical systems of complex interactions (Newman, 2014). Learning the structural form and the parameters of a dynamical system allows one to predict not only the evolution of the system but also the effects of manipulation and perturbation. Depending on the characteristics of the biological system under study as well as on the available measurements, a palette of dynamical model formalisms has been successfully applied. For deterministic processes typical types of models include difference equations (when time is modeled as discrete), ordinary differential equations (ODEs) for continuous time and their space-extended counterpart, partial differential equations. For stochastic processes, typical types of models include Markov Chains such as auto-regressive and moving averages (for discrete time), and, stochastic differential equations for continuous time. Due to the absence of a unifying mechanism, inferring the structure and the parameters of a dynamical system depends on the underlying formalism requiring specialized techniques and algorithms.

Several approaches that learn the structural form of an ODE system have been presented in the literature. One of the first attempts was the Inferelator (Bonneau *et al.*, 2006) which is an algorithm for *de novo* learning of parsimonious regulatory networks from systems-biology data sets using a shrinkage operator that induces sparsity. ODEion (Gennemark and Wedelin, 2014) searches over the model space and performs an optimization via simulation approach while SINDy is another sparsity-induced algorithm (Brunton *et al.*, 2016; Mangan *et al.*, 2016). Sparse regression dedicated on single-cell data have been also proposed (Klimovskaia *et al.*, 2016). Generally, sparse solutions are obtained through convex relaxation approaches (Tropp, 2006) such as linear programing or Lasso (Tibshirani, 1994) and, not surprisingly, such optimization programs have been studied extensively in structure inference and network reconstruction (August and Papachristodoulou, 2009; Bolstad *et al.*, 2011; Charbonnier *et al.*, 2010; Friedman *et al.*, 2008; Gustafsson *et al.*, 2009). Bayesian inference approaches (Daniels and Nemenman, 2015; Friston *et al.*, 2003) have been also utilized for structure learning and phenomenological modeling of deterministic dynamical systems. Bayesian methods can tackle unmeasured (latent) variables on the cost of increased computational demands due to the substantial sampling of the model space. Moreover, theoretical guarantees on the performance are hard to obtain for Bayesian methods. Studies on stochastic dynamical systems prove under appropriate assumptions that the true sparse stochastic dynamics is guaranteed to be inferred (Bento *et al.*, 2010; Bolstad *et al.*, 2011). The prominent feature of these studies is that the dynamical system is linear with respect to both the unknown parameters and the state variables. Given the structure of the dynamical system, parameter estimation algorithms are also partitioned according to the type of the dynamical system (DiStefano, 2015).

In this paper, we present Unified Sparse Dynamics Learning (USDL) algorithm which is a novel approach to infer both the structure and the parameters of any dynamical system from temporal measurements. First, by employing the *weak formulation* (Davis, 1984; Strang and Fix, 2008), the problem of inducing the structure of a dynamical model is transformed into an equivalent yet atemporal learning problem. The weak formulation can be intuitively understood as a projection operator that multiplies the dynamical system’s equation by an arbitrary function, called a test function, and, then integrates over time and/or space. The weak formulation can be also thought as a type of feature extraction. The weak formulation has several advantages: (i) by using integration-by-parts, the weak formulation does not require the computation of the derivatives of the trajectories; in contrast, other methods compute numerically the derivatives thus amplifying the noise and deteriorating the reconstruction accuracy, (ii) by suitable definition of the test functions, the same algorithm can be applied to almost any type of dynamical system, thus, *unification* across different families of dynamical models is achieved and (iii) the weak formulation transforms the dynamical system into a *linear system of equations* where the time and/or space dimensions have been completely eliminated.

Second, we assume sparsity of the solution which results—in combination with the weak formulation—into a well-posed, well-studied problem in computer science, namely sparse signal recovery (SSR) (Bruckstein *et al.*, 2009; Candès *et al.*, 2006) also known as compressed sensing (Donoho, 2006; Foucart and Rauhut, 2013). Sparsity in our context means that the dynamics of each state variable are typically driven by a relatively small number of variables. Sparsity is critical for learning large systems from finite data and constitutes a form of complexity penalization and regularization thus favoring simpler solutions. The reformulation of the problem as a SSR problem allows us to straightforwardly apply a large vividly evolving body of theoretical and algorithmic results. Specifically, we choose the Orthogonal Matching Pursuit (OMP) algorithm (Davis *et al.*, 1997; Pati *et al.*, 1993; Tropp and Gilbert, 2007) which is a greedy and fast algorithm for recovering the sparse solution while theoretical guarantees on the correctness of the learned solution are provided based on the mutual incoherence parameter (MIP) (Donoho and Huo, 2001) and the exact recovery coefficient (ERC) (Tropp, 2006). The presented examples reveal that the above (in)coherency metrics and especially the latter are informative indicators of the reconstruction accuracy as measured by the precision and recall curves. Furthermore, multiple interventions typically convey crucial information about the true structure of a biological dynamical system (Sachs *et al.*, 2005). The proposed USDL algorithm is capable of handling not only observational but also interventional data, a property that distinguishes it from the existing sparsity-induced approaches.

Finally, we compare USDL algorithm with SINDy (Brunton *et al.*, 2016) and despite the fact that SINDy occasionally converges faster than USDL with respect to the amount of data as in Lorenz96 model (Lorenz, 1996), we demonstrate that our approach performs better in terms of accuracy in both interventional time-course data and stationary stochastic time-series as shown in the Section 3 by the respective protein networks and multidimensional Ornstein–Uhlenbeck process given sufficient amount of temporal data. The merit of the weak formulation is notably highlighted at the stationary regime of the Ornstein–Uhlenbeck process where an educated guess of test functions resulted in perfect reconstruction of the stochastic dynamical system showing the plasticity and generality of USDL algorithm which stems from the plethora of choices for the test functions.

## 2 Materials and methods

### 2.1 Weak formulation

The weak formulation has been employed primarily in the field of applied mathematics where it provides a rigorous theoretical framework to define solutions that are not necessarily differentiable (Evans, 1998). Another important application of the weak formulation is found in numerical analysis. Particularly, the finite elements method which is a numerical technique for estimating approximate solutions of dynamical systems is based on it (Strang and Fix, 2008). In our setting, the solutions are given as measurements while the system of differential equations is unknown and has to be inferred. Thus, instead of applying the weak formulation to approximate solutions, we use it to transform the problem of learning the structure of a dynamical system into an equivalent yet atemporal learning problem.

For clarity purposes, the weak formulation is presented for ODEs which is a particular example of dynamical systems. Let $x=x(t)\in R^{N}$ be an *N*-dimensional vector function of time which represents the state variables while *N* is the number of state variables. Following physics notation (i.e. $\dot{x}:=\frac{dx}{dt}$), a system of ODEs with linear parameters is defined as $\dot{x}=A\psi (x),\;x(t_{0})=x_{0}$ where *t*_0_ is the starting time instant with initial value $x_{0}\in R^{N}$. $A\in R^{N\times Q}$ is the unknown and usually sparse connectivity (or coefficient or parameter) matrix to be estimated. Dictionary, ψ(·), is a (given) *Q*-dimensional vector-valued vector function, $\psi :R^{N}\to R^{Q}$ which contains all the pre-determined candidate functions that might drive the dynamics. Candidate functions are usually powers, cross-products, fractions, trigonometric, exponential or logarithmic functions of the state variables leading to non-linear dynamical systems. The completion of the dictionary, which could be guided by the presented incoherence metrics, is typically an application- and/or user-specific problem. Element-wise, the ODE system can be equivalently rewritten in a non-matrix form as

(1) $${\dot{x}}_{n}=\sum_{q=1}^{Q}a_{nq}\psi_{q}(x)\;,\;x_{n}(t_{0})=x_{0n}\;,\;n=1,...,N.$$

An example from the theory of biochemical reaction networks (Lente, 2015) is presented in Table 1. Under mass action kinetics law and depending on the number of reactants in a chemical reaction network, two candidate dictionaries are shown. If the user assumes that only single-reactant reactions occur then the dictionary is simply the identity function (i.e. ψ(x)=x) since the reaction rates are linear with respect to the state variables for this case. However, if the user assumes that two-reactant reactions occur then the dictionary is augmented with all quadratic terms as shown in the last row of Table 1.

**Table 1.** Different choices for the dictionary, ψ(x), according to the allowed chemical reaction types under mass action kinetics law

Unknown reactions	Dictionary, ψ(x)	Size, *Q*
$X_{i}\to X_{i\prime }$	*x*	*N*
$X_{i}+X_{j}\to X_{i\prime }+X_{j\prime }$	${\left[x,x_{1}x_{1:N},x_{2}x_{2:N},...,x_{N}^{2}\right]}^{T}$	(N+3)N/2

*Note*: Symbols *X*_1_–*X_N_* correspond to the state variables [a.k.a. (reaction) species in chemistry and systems biology]. The row vector $x_{i:j}$ is defined as $x_{i:j}:=[x_{i},x_{i+1},...,x_{j}]$. For the two-reactant case, the dynamical system is non-linear with respect to the state variables.

In order to derive the (finite) weak formulation, a set of *M* test functions denoted by ${\left\{\varphi_{m}(t)\right\}}_{m=1}^{M}$ has to be specified. The test functions are smooth, not necessary orthogonal and can be chosen from a large repository of functions. Typical examples are polynomials, splines, Fourier modes (i.e. sines and cosines with varying frequency) or kernel functions from other integral transforms. Fourier modes are preferred when trajectories exhibit periodicities while splines are more appropriate when localized phenomena have to be highlighted. As we will show later, customized families of test functions may be required in order to reveal imperceptible variable interactions. Proceeding, let *T* be the final time and without loss of generality assume that $t_{0}=0$. Denoting by $\langle f,g\rangle :=\int_{0}^{T}f(t)g(t)dt$ the inner product between two functions *f* and *g* belonging to the *L*^2^ function space, define the *M*-dimensional vector *z_n_* whose *m*-th element is given by $z_{nm}:=\langle {\dot{x}}_{n},\varphi_{m}\rangle$, the *M* × *Q* dictionary matrix Ψ whose (*m*, *q*)-th element is given by $\Psi_{mq}:=\langle \psi_{q}(x),\varphi_{m}\rangle$ and let *a_n_* be a *Q*-dimensional vector which corresponds to the *n*-th row of matrix *A.* Then, the weak form of the ODE system in equation (1) is

(2) $$z_{n}=\Psi a_{n}\;,\;n=1,...,N.$$

In Supplementary S1 Text, we provide the detailed derivation of the weak formulation for ODEs as well as for other types of dynamical systems.

The intuition behind weak formulation is that it projects the solution of a dynamical system to a finite-dimensional vector (i.e. to a set of linear functionals in mathematical language) whose elements are defined from the inner product between the solution and the test functions. A key advantage of the weak formulation is that appropriate test functions for various dynamical systems such as partial and/or stochastic differential equations exist and can be utilized transforming again the dynamical inference problem to an atemporal/aspatial problem similar to equation (2). Thus, *unification* of the structure inference problem for various dynamical systems is achieved. Moreover, the original problem where time is continuous is transformed from an infinite dimensional [meaning that equation (1) should be satisfied for all t∈[0,T]] to a finite dimensional one. Additionally, and more importantly from a practical viewpoint, *there is no need to numerically estimate the time derivatives of the state variables.* Indeed, exploiting the integration-by-parts formula, it is straightforward to obtain that

$$z_{nm}=x_{n}(t)\varphi_{m}(t){|}_{0}^{T}-\langle x_{n},{\dot{\varphi }}_{m}\rangle ,$$

and since test functions, $\varphi_{m}$, are explicitly know, their differentiation is exact. In general, differentiation amplifies the noise of a signal resulting in high variance estimates of the derivatives deteriorating the performance of any inference approach which necessitates the use of derivative approximation and regularization. Finally, when new trajectories (or time-series) from different initial conditions are obtained, they can be easily incorporated into the formulation by straightforward concatenation. Indeed, if *P* trajectories, ${\left\{x^{\left(p\right)}(t)\right\}}_{p=1}^{P}$, are provided and expanding both

$$z_{n}=\left[\begin{array}{c} z_{n}^{\left(1\right)}\\ \vdots \\ z_{n}^{\left(P\right)} \end{array}\right]\in R^{MP}\text{ and }\Psi \text{=}\left[\begin{array}{c} \Psi^{\left(\text{1}\right)}\\ \vdots \\ \Psi^{\left(\text{P}\right)} \end{array}\right]\in R^{\text{MP}\times \text{Q}}$$

with $z_{nm}^{\left(p\right)}=\langle {\dot{x}}_{n}^{\left(p\right)},\varphi_{m}\rangle$ and $\Psi_{mq}^{\left(p\right)}=\langle \psi_{q}(x^{\left(p\right)}),\varphi_{m}\rangle$, then, equation (2) is still valid.

### 2.2 Type of measurements

In the weak formulation, the estimation of the integrals (i.e. the inner products between functions) from the temporal data is required. There are two major categories of temporal data that we consider depending whether the same object is repeatedly measured or not. For the case of repeated measurements, the same object is measured sequentially over a time interval hence a time-series is constructed at the sampling points. When the sampling frequency is high enough, the collected time-series can be considered as continuous over time. For repeated measurements and deterministic systems, standard techniques such as trapezoidal rule and Simpson’s rule are utilized for the numerical integration. When measurements are far from each other, interpolation between the time-points can be applied. Additionally, there is no need for equispaced sampling since these methods can handle uneven sampling. For stochastic integrals, numerical integration requires different treatment since the definition of the integrals is different (e.g. Ito integral), nevertheless, numerical methods do also exist for this case (Oksendal, 1985).

Non-repeated measurements—we also refer to them as time-course data—measure at each time instant a different object. This may happen because the object is destroyed during the measurement process as, for instance, in mass cytometry (see the demonstration examples below) and the same object cannot be measured more than once. However, it is assumed that all the measured objects are drawn from the same distribution. For time-course data, time-series cannot be directly constructed from the data. In order to create time-series from the time-course data, the collocation method (Ramsay *et al.*, 2007) in conjunction with a trajectory smoothing penalty (Craven and Wahba, 1978; Zhan and Yeung, 2011) are utilized. In the collocation method, a time-series is approximated by a weighted sum of basis functions.

Moreover, time-course data from different experiments might be available. Each experiment might perform interventions to some or all variables hence each variable has its own time-series which resembles the so called multiple shooting method (Peifer and Timmer, 2007) and therefore each variable has its own trajectory per experiment. Interventional data are important in structure inference because they often reveal critical information about the biological system under study. Details on the collocation method and how to deal with interventions (e.g. inhibition) using the multiple shooting method can be found in Supplementary S2 Text and S3 Text, respectively.

### 2.3 Sparse signal recovery

The weak formulation enables us to transform a dynamical system inference problem to the identification of the sparse solution of equation (2) which belongs to the well-studied field of SSR. In particular, the structure inference is transformed to the problem of finding the support of the sparse solution. In SSR, the goal is to minimize the *l*_0_ quasi-norm of the coefficient vector, *a*, given that $||z-\Psi a|{|}_{2}<\text{ε}$ where *ε* is a pre-defined tolerance (for the sake of simplicity, we drop the dependence on *n* for the rest of the section.). Performing the minimization is computationally feasible in small scale systems but, in general, it is an intractable problem since it grows exponentially, hence, alternative approximation methods have been developed. One type of approximation is based on the so-called convex relaxation approach where the above optimization is replaced by a convex program (Tropp, 2006). Convex relaxation is appealing because the optimization can be completed in polynomial time using standard software. General conditions under which the convex relaxation program returns the right answer has been presented in the literature (Tropp, 2006). In the presence of noise, additional assumptions are imposed on the strength of the components’ coefficient in order to achieve perfect reconstruction with high probability. In Supplementary S4 Text, we present two commonly used convex relaxation formulations namely *l*_1_ error where instead of minimizing the *l*_0_ norm, the *l*_1_ norm is minimized and Lasso where an *l*_1_ norm regularization term is added to the quadratic cost functional. Another family of techniques that solves the SSR problem is greedy algorithms such as matching pursuit (Mallat and Zhang, 1993) and OMP (Davis *et al.*, 1997; Pati *et al.*, 1993; Tropp and Gilbert, 2007). For these greedy algorithms, the correct support of the signal is recovered under suitable assumptions which are similar to the assumptions of convex relaxation. Details on OMP as well on known theoretical results that assert under which conditions these algorithms infer the true sparse representation are provided in Supplementary S4 Text.

In order for any sparse signal identification algorithm to perform perfect reconstruction both the degree of collinearity among the columns of Ψ and the signal-to-noise ratio have to be properly controlled. MIP first introduced in Donoho and Huo (2001) which is defined by

(3) $$\mu (\Psi ):=\underset{1\leq q,q\prime \leq Q,q\neq q\prime }{\max}\frac{\left|\psi_{q}^{T}\psi_{q\prime }\right|}{||\psi_{q}|{|}_{2}||\psi_{q\prime }|{|}_{2}}$$

is a measure of similarity between the columns of Ψ. In the extreme cases, μ(Ψ)=0 when the matrix is orthogonal while μ(Ψ)=1 when for instance there are two columns of the matrix which are collinear. In Supplementary S4 Text, theorems that determine under which conditions on MIP the SSR algorithms are guaranteed to return the correct solution are presented. However, MIP can be unimportantly conservative because it penalizes for the correlation of features that may not participate in the solution. Indeed, if two columns of Ψ are highly similar (i.e. collinear or strongly dependent) but not part of the solution then MIP is close to one but the SSR algorithms are still expected to estimate the right solution. This can be circumvented with ERC which does not measure the collinearity between two columns in general but measures only the collinearity of the subspace defined by the solution with respect to each column not in the solution. The definition of ERC given a set of indices, S⊂{1,...,Q} (Tropp, 2004), is

(4) $$\text{ERC}(\text{S}):=1-\underset{q\prime \notin \text{S}}{\max}||{\left({\overset{¯}{\psi }}_{\text{S}}^{T}{\overset{¯}{\psi }}_{\text{S}}\right)}^{-1}{\overset{¯}{\psi }}_{\text{S}}^{T}{\overset{¯}{\psi }}_{q\prime }|{|}_{1}$$

where ${\overset{¯}{\psi }}_{q}:=\frac{\psi_{q}}{||\psi_{q}|{|}_{2}}$ are the normalized dictionary atoms (i.e. normalized columns of Ψ) while ${\overset{¯}{\Psi }}_{\text{S}}$ is the matrix that contains only the columns of $\overset{¯}{\Psi }:=[{\overset{¯}{\psi }}_{1}|...|{\overset{¯}{\psi }}_{Q}]$ that are indexed by the set S. Letting T be the true index set (i.e. the support of the true signal, or, in our case, the true structure of the dynamical system), a necessary condition for the convex relaxation algorithms or for the OMP to correctly solve SSR is ERC(T)>0. The difficulty for estimating ERC arises from the fact that the true index set, T, is not known a priori. Nevertheless, it can be used as a posteriori indicator of accuracy. Under noise, OMP algorithm with the standard stopping criterion returns the true solution, if it additionally holds that (Cai and Wang, 2011)

(5) $$|a_{q}|\geq \frac{2}{\text{SNR}(q)ERC(T)\lambda_{\min}(T)}\;,\text{ for all }q\text{ =1},...,Q$$

where $\text{SNR}(q)=\frac{||\psi_{q}|{|}_{2}}{||e|{|}_{2}}$ is the signal-to-noise ratio between the *q*-th dictionary atom and the error/noise vector e:=z−Ψa while $\lambda_{\min}(T)$ is the smallest eigenvalue of the matrix ${\overset{¯}{\Psi }}_{T}^{T}{\overset{¯}{\Psi }}_{T}$. Overall, *monitoring these quantities is extremely informative on determining the quality and the confidence of the learned structure.*

**Remark 1:**

There is a difference between our sparse identification problem and typical SSR. In standard SSR, the number of dictionary atoms (columns of Ψ) is usually larger than the number of measurements (rows of Ψ) while the opposite is true here since multiple experiments and multiple interventions are typically performed and measured.

**Remark 2:**

SSR algorithms have one hyperparameter that needs to be fine-tuned. We incorporate a selection process using the *F*1 score, which is the harmonic mean of precision and recall, as a performance metric. We always select the value that maximizes the *F*1 score.

**Remark 3:**

Restricted isometry property (Candes and Tao, 2005) like MIP is another a priori metric that ensures perfect reconstruction, however, like MIP, it suffers from the same problem of being too conservative. Additionally, it is computationally expensive thus we choose not to present it in detail.

### 2.4 Algorithmic summary

Before proceeding with the demonstration examples, a coarse summary of the proposed dynamical system inference algorithm is presented below as pseudo-code.

**Algorithm 1: USDL**

1: **Input:** Time-series or time-course measurements, dictionary, ψ(x), and set of test functions, $\left\{\varphi_{m}\right\}$.

2: **if** time-course measurements **then**

3: Apply the Collocation method.   ▹ Time-series interpolation

4: Compute Ψ and $z_{n},\;n=1,...,N$.  ▹ Weak formulation

5: Estimate MIP from Ψ.

6: **for**n=1,...,N**do**        ▹ For each row of *A*

7:  ${\hat{a}}_{n}=\text{SSR}(z_{n},\Psi )$        ▹ Solve SSR problem

8:  Estimate $ERC({\hat{T}}_{n})$.

9: **Output:**$\hat{A}$, MIP and ERC.

## 3 Results

The inference capabilities of the proposed approach for several classes of dynamical systems are presented. Source code that produces the figures is available (Supplementary S1 Code). Among the algorithms that are capable of solving SSR, we adopt OMP due to the following reasons. First, it is computationally more efficient since a forward selection of the components is performed. Second, the theoretical justifications between the various methods are very similar making the algorithms almost equivalent. Third, the hyperparameter of OMP is more intuitive compared for instance with Lasso since it is the energy of the noise term (i.e. $||e|{|}_{2}$). Indeed, we approximate the noise energy as (1+α) times the *l*_2_-norm of the residual between the signal and the complete Least Squares solution with *α* being usually a small positive number. OMP stops when relative residual energy becomes smaller than *α*, therefore, OMP returns sparser solutions as we increase the value of *α* (see Supplementary S4 Text for more details). Lastly, it is straightforward to incorporate prior knowledge by adding any known contribution to the dynamics, hence, it is straightforward to include data with one or more interventions with known effects.

### 3.1 Protein interaction network

**Fig. 1.**
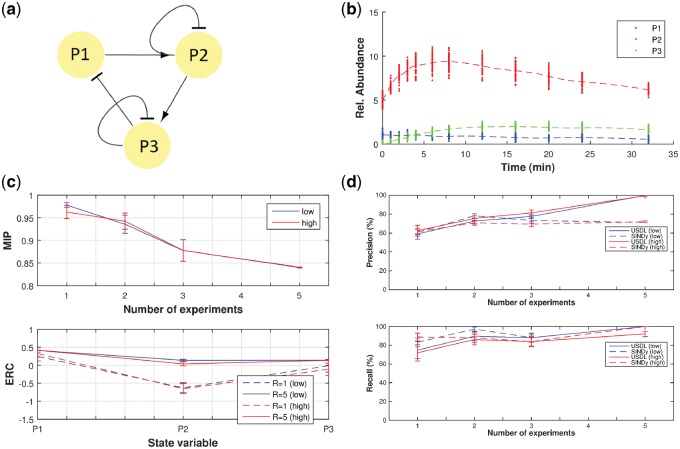
(**a**) The network of interactions between the three species (P1, P2 and P3). This graph is a coarse high-level representation and it should not be confused with the detailed biochemical reaction network which is given in Supplementary S5 Text. (**b**) Time-course measurements (dots) and the estimated smoothed trajectories (dashed curves). The collocation method in conjunction with smoothing penalty is used for the estimation of the time-series. Notice that time-course data from the low noise case are shown. (**c**) MIP and ERC for P1–P3 as a function of the number of experiments under low (blue) and high (red) measurement uncertainty. Standard deviation of the various stochastic terms in high uncertainty regime is twice as much compared to the low uncertainty regime. From the lower plot, it is evident from the negativity of ERC that the problematic variable is P2 when only one experiment is used. (**d**) Precision and recall curves under low (blue) and high (red) measurement noise as a function of the number of experiments. Results from both USDL (solid lines) and SINDy (dashed lines) algorithms are presented. Perfect inference is achieved only with USDL under five experimental interventions and the low noise case. Precision (solid lines) seems to be insensitive to higher noise levels, however, recall slightly degrades

The first demonstration is a simulation of mass cytometry measurements with a three-species prototypical protein interaction network with cycles. The complete biochemical reaction network is given in Supplementary S5 Text and it has been simulated with standard ODE solver. The ground truth of interactions are shown in Figure 1a where the arrow means that the source variable up-regulates the target variable while the vertical bar means that the source variable down-regulates the target variable. The experimental setup assumes that P1, P2 and P3 are measured at specific time-points and every sample is destroyed during the measurement (see dots in Fig. 1b). In order to apply the weak formulation, time-series have to be constructed, thus, we first apply the collocation method with smoothing penalty. Dashed curves in Figure 1b correspond to the estimated time-series. If, additionally, multiple experimental interventions are performed, the multiple shooting method is applied and one time-series per experiment is obtained.

Proceeding, despite the fact that the complete biochemical reaction network is non-linear (As explained in Supplementary S5 Text, the complete reaction network is unfortunately unidentifiable making it an improper example for testing the learning algorithms.) with respect to the state variables, the assumed model for inference is linear,

$$\dot{x}=Ax\;,$$

where vector $x(t)\in R^{3}$ contains the abundance of P1–P3 at time instant *t.* Connectivity matrix, *A*, encodes the direct causal interactions within the set of state variables. Indeed, if element *a_nq_* is zero then no direct causal interaction exists from species *x_q_* to *x_n_.* If *a_nq_* is positive then an increase of variable *x_q_* implies an increase of the rate of *x_n_* which results in increasing the concentration of *x_n_* thus *x_q_* activates or up-regulates *x_n_* and it is denoted with an arrow from *x_q_* to *x_n_.* On the contrary, if *a_nq_* is negative then *x_q_* inhibits or down-regulates *x_n_* and it is denoted with a vertical bar. Thus, the structure of the network can be induced from the matrix *A.* Indeed, both the strength and the type of an interaction is inferred from the absolute value and the sign of the corresponding element of *A*, respectively. Furthermore, it is noteworthy that several sources of error exist in this benchmark example. First, there is measurement error related to the machine limitations and it is assumed to be additive. Second, there is uncertainty error due to the fact that each measurement comes from a different cell and each cell has different concentrations of the measured quantities as Figure 1b demonstrates. Additional sources of error stems from the facts that (i) the complete reaction network is non-linear for the state variables while the assumed model is not and, (ii) not all species are measured since the compound P1P3 is not quantified resulting in the existence of latent confounding variables.

Figure 1c presents the MIP as a function of the number of experiments (upper plot) as well as the ERC for each state variable (lower plot). Even though MIP drops as the number of experiments is increased, the decrease is not significant making the correct inference of the interaction network a priori less certain. ERC which is a posteriori metric, asserts that the problematic variable is P2 when only one experiment is fed to the inference algorithm (dashed lines in lower plot of Fig. 1c) while ERC is positive when all five interventions are used for the inference making one crucial assumption for perfect reconstruction true. However, this is not always enough as shown be the performance of the algorithms when the measurement noise is doubled (red solid lines in Fig. 1c and d).

Indeed, the precision-recall curves of Figure 1d assert that the network is partially reconstructed when USDL (solid lines) fed with data from only one intervention is applied. In contrast, the true network is reconstructed when all five interventions are taken into account. Note that 41 Fourier modes which correspond to a constant function, 20 sines and 20 cosines were used as test functions in USDL algorithm. Additionally, Figure 1d presents the reconstruction accuracy for the SINDy algorithm (dashed lines). The hyperparameter value for both approaches is optimally selected by maximizing the *F*1 score which is shown in Supplementary Figure S1a of S5 Text. When one intervention is used, the performance of SINDy is slightly better to USDL, however, SINDy does not improve its accuracy as the number of experiments increases. Evidently, SINDy is not capable of handling datasets that have multiple complex interventions. Moreover, we evaluate the forecast capabilities of the inferred model on new experiments. Prediction accuracy (Supplementary Fig. S1b in S5 Text) is in accordance with the precision-recall curves (i.e. Fig. 1d) in all cases revealing once again that the problematic variable is P2. Overall, this demonstration example shows the necessity of designing and executing several experiments for guaranteed perfect reconstruction of a network from non-repeated time-course measurements. In Supplementary S5 Text, we present additional case studies on the performance of the proposed approach when the number of sampling points is reduced as well as when different weights for the smoothing penalty in the collocation method are applied.

**Remark 4:**

For small systems, a brute force alternative is tractable. A complete search of all possible solutions when the non-zero components are less than ten is computationally feasible for dictionary size up to twenty atoms. However, such an approach will provide little or no information on how to design a new experiment or a new data acquisition policy compared with greedy algorithms or convex relaxation methods where metrics such as MIP and ERC can guide the experimental designer.

### 3.2 Protein network inference from mass cytometry data

The second demonstration is the inference of protein interactions from publicly available mass cytometry data (Krishnaswamy *et al.*, 2014). Single-cell analysis and particularly mass cytometry widely opens new directions for understanding cellular responses to perturbations and cellular functionalities due to the capability of measuring tens of proteins in each cell. Moreover, it can be multiplexed resulting in studying the cells under different conditions and time-points in a relatively cheap and fast way (Bodenmiller *et al.*, 2012; Krishnaswamy *et al.*, 2014). Given the high resolution of single-cell analysis it is expected to become a standard technique in medical sciences in the near future. In Krishnaswamy *et al*. (2014), 13 time-points are sampled and measurements are separated into 3 subpopulations, namely, CD4+, CD8+ and Effector/Memory. Two activation cocktails which stimulate the receptors CD3/CD28 and CD3/CD28/CD4 were applied, respectively. Each experiment was repeated twice with different activation levels. Reconstructing the signaling pathway upon activation is a non-trivial task because few proteins inside the cells are measured and on top of that many interfering mechanisms with different rate are also occurring. Both result in a large number of latent confounding factors. Thus, it is very hard to reconstruct directly the complete system of interactions. However, network reconstruction would be more successful if restricted to subnetworks.

**Fig. 2.**
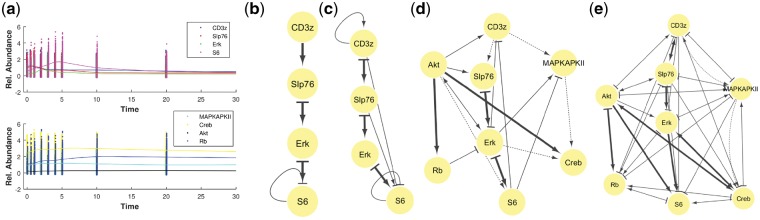
Network reconstruction of protein interactions from temporal mass cytometry data. (**a**) Time-course measurements (dots) and the estimated smoothed trajectories (solid lines). The collocation method in conjunction with smoothing penalty was used for the estimation of the time-series. Observe the high level of stochasticity of the time-course mass cytometry data. (**b**) The reconstructed subnetwork with four proteins using the USDL algorithm. Bold arrows (true positives) indicate that the true network of interactions is inferred. (**c**) Similar to (b) for SINDy. (**d**) The reconstructed network with eight proteins with non-bold arrows corresponds to false positives while dotted arrows correspond to false negatives. Dynamics for the additional proteins vary less over time as it is evident from the lower panel of (a). Nevertheless, most of the interactions are directly (such as Akt → Rb or Erk → S6) or indirectly (like Akt → Erk → S6 instead of Akt → S6) inferred. (**e**) Similar to (d) for SINDy

We perform network inference for two subnetworks with the first being a cascade of CD3z, SLP76, Erk and S6 proteins while the second is enriched with MAPKAPKII, Creb, Akt and Rb. Figure 2a presents the trajectories of the proteins estimated from the mass cytometry data using the collocation method with smoothing penalty. The multiple shooting method is utilized for each subpopulation and each experiment. We note that the collocation method assumes that the overall measurement noise is Gaussian, however, we observed that the noise in the mass cytometry data is sometimes skewed and/or multimodal potentially deteriorating the quality of the estimated trajectories. Furthermore, time-series adjustment is performed by subtracting the minimum value of each trajectory which merely corresponds to the state of no activity. As it is evident from the figure, the level of stochasticity is high making the dense sampling of the signaling phenomenon necessary.

Figure 2b and c presents the reconstruction of the smaller subnetwork for USDL and SINDy algorithm, respectively, while Figure 2d and e presents the reconstruction results for the larger subnetwork. We consider a linear ODE model ($\dot{x}=Ax$) excluding more complicated protein interactions. USDL algorithm utilizes 21 Fourier modes while the hyperparameter for each approach is fine-tuned based on the *F*1 score estimated on an independent subset of the data (see Supplementary Fig. S6 in S5 Text) using the KEGG database (Kanehisa and Goto, 2000) as ground truth. The semantics of arrow and bar edges are the same as in the previous example. An edge is bold when it is also found in the KEGG database, regular if found with USDL (or SINDy) but it is not found in KEGG database while it is dotted if it is in the KEGG database but not found. Inference is repeated 100 times using a portion of the available data in each iteration and the reported edges are the ones that are found at least in 80% of the times. Concentrated in the case with four proteins, the subnetwork is correctly reconstructed with USDL algorithm while SINDy infers two additional edges. The bar edges in Figure 2b, which imply down-regulation, are explained as a mechanism to model the degradation of each variable over time. When four more proteins are added, the network reconstruction becomes harder. Nevertheless, USDL using CD4+ subpopulation and CD3/CD28 activator (see Fig. 2d) was able to recover half of the known edges. The cascade Slp76 → Erk → S6 is still correctly inferred. However, CS3z was replaced by Akt in the phosphorylation of Slp76. Additionally, the proposed algorithm correctly assesses the influence of Akt to the phosphorylation of both Rb and Creb showing that Akt plays a central role in pathway signaling of T cells. In contrast, SINDy with the optimal hyperparameter returns an almost fully-connected graph resulting in a non-sparse solution.

The difficulty in inferring the KEGG-based network of protein interactions is reflected on both MIP and ERC (reported in Supplementary S5 Text). Both incoherency metrics deteriorate when more proteins are added to the analysis making the assumptions for perfect reconstruction less valid. Thus, more experiments are required in order to improve the accuracy of the network inference algorithm. These experiments can be guided by metrics such as MIP or ERC. Additional demonstrations can be found in Supplementary S5 Text. Particularly, the reconstructed networks when additional activators and/or subpopulations are considered are presented as well as how important is the high sampling rate for protein interactions inference through mass cytometry data. For instance, the reconstruction accuracy is severely reduced when removing half of the time-points as it is shown in Supplementary S5 Text. Finally, we have integrated the USDL algorithm in SCENERY (http://scenery.csd.uoc.gr/) (Papoutsoglou *et al.*, 2017) which is a web tool for single-cell cytometry analysis. SCENERY provides a comprehensive and easy-to-use graphical user interface where users may upload their data and perform various types of protein network reconstruction. The incorporation of USDL algorithm into SCENERY aims to increase its reusability and, hopefully, its popularity.

### 3.3 Multidimensional Ornstein–Uhlenbeck process

The last example is a multidimensional Ornstein–Uhlenbeck process which is a system of linear stochastic differential equations with additive noise. It has applications in evolutionary biology where multiple traits are modeled over time with multidimensional Ornstein–Uhlenbeck process (Bartoszek *et al.*, 2012) as well as in particle physics (Gardiner, 2004) and finance (Gardiner, 2009). Mathematically, the driving system of equations is given by

(6) $$\dot{x}=-Ax+\sigma \dot{B}\;,$$

where $x(t)\in R^{N}$ is the stochastic process, connectivity matrix $A\in R^{N\times N}$ determines the interactions between the variables, σ∈R corresponds to the noise level while $B(t)\in R^{N}$ is an *N*-dimensional standard Brownian motion. Intuitively, the derivative of a Brownian motion can be understood as a continuous-time zero-mean white noise with variance one. We set *N *=* *20 while matrix *A* is defined as the graph Laplacian with random edges and maximum outgoing degree =3.

**Fig. 3.**
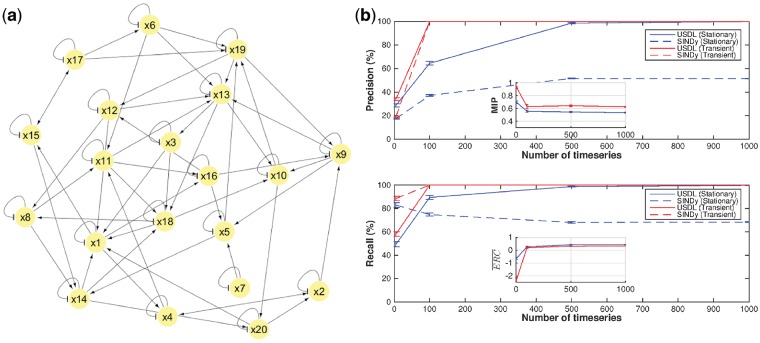
Performance analysis and comparison between USDL and SINDy algorithms for Ornstein–Uhlenbeck stochastic process. (**a**) The connectivity graph for each variable of the Ornstein–Uhlenbeck process. The edges, their direction as well as the type of interaction are determined by the non-zero elements of connectivity matrix *A.* (**b**) Precision and recall are shown as functions of the number of measured time-series in two different regimes; stationary (blue) and transient (red). Both USDL (solid) and SINDy (dashed) algorithms achieve perfect reconstruction of the dynamical system for the transient regime and when enough time-series are measured. For the stationary regime, perfect reconstruction is possible only for USDL and a special type of test functions (peaky Fourier modes) while SINDy (blue dashed) fails to recover completely the dynamical system in this regime due to the high stochasticity

Figure 3a presents the graph of interactions for each variable of a randomly drawn instance of *A.* We distinguish between two regimes, namely, the stationary (or equilibrium) regime and the transient regime. At stationarity, the driving force is primarily the stochastic or diffusion term [second summand in the r.h.s. of equation (6)] with the deterministic or drift term [first summand in the r.h.s. of equation (6)] acting as a stabilizer. In the transient regime, the dynamics are primarily driven by the drift term. This separation is of great importance because the signal in the former case is buried under the noise (i.e. both have approximately the same energy or, in other words, the signal-to-noise ratio is ∼1) while the signal is stronger compared to the stochastic term in the transient regime (see the simulated trajectories for both regimes in Supplementary Fig. S12a). As performance measures show in Figure 3b, both USDL and SINDy are able to infer the correct structure of *A* (i.e. the correct graph of interactions) at the transient regime (red curves) when enough—approximately *P *=* *100—time-series are provided. We remark here that, as in the previous examples, the hyperparameter values of both algorithms are selected based on the maximum *F*1 score. Averaged ERC is positive for this setup as inset plot reveals and signal-to-noise ratio is high enough to theoretically guarantee the perfect reconstruction of the dynamical system.

In the stationary regime, the driving force is the noise and positive (averaged) ERC is not enough to guarantee perfect reconstruction and control of the signal-to-noise ratio is required for true structure learning. We tested a series of typical test functions such as Fourier modes and splines as well as we varied the number of test functions, however, we were not able to perfectly reconstruct the dynamical system with the accuracy hitting a plateau (figures in Supplementary S5 Text) despite the fact that theoretical results (Bento *et al.*, 2010) suggests that true recovery is possible. The problematic cases arise from variables whose time cross-correlations have similar shape and they are close to each other thus we need to define another type of test functions with the property of having sharp changes which assist the separation between small time-differences. A well-educated choice of test functions, which we named peaky Fourier modes (details in Supplementary S5 Text) result in perfect reconstruction of the connectivity matrix for the stationary regime when USDL is applied (blue solid lines in Fig. 3b). In contrast, SINDy algorithm (dashed blue lines) is incapable of inferring the structure of the dynamical system because it is not designed for stochastic systems and thus it is unable to handle the high intensity of the noise which is observed in the stationary regime.

Moreover, we measured the root mean squared error (RMSE) on the elements of the connectivity matrix *A* and assess the parameter estimation behavior of the inference approaches. RMSE results (Supplementary Fig. S13b in S5 Text) reveal that two orders of magnitude lower RMSE is reported in the transient regime relative to the stationary regime. In the same figure, it is shown that the RMSE for USDL is lower than the RMSE for SINDy which is in accordance with the structure inference performance (i.e. the precision-recall curves).

## 4 Discussion

The weak formulation enables the transformation of any spatio-temporal dynamical system that is linear with respect to its parameters into a linear system of equations. The unification through the weak formulation creates the foundations for general dynamical system inference in biological applications. For instance, in mass cytometry and more generally in single-cell analysis, not trajectories but the distribution of the species populations is measured over time. Thus, the structure learning from partial differential equations such as the Fokker–Planck or the master equation (Gardiner, 2004) which both describe the evolution of the probability distribution of the measured quantities can be transformed into a linear set of equations. Moreover, the avoidance of differentiation through the integration-by-parts trick could benefit the already existing dynamical inference algorithms, especially, in adverse, noisy conditions. Additionally, the transformed structure learning problem can be considered not only as an SSR problem but also as a feature selection problem (Guyon *et al.*, 2003), a subfield of machine learning and statistics. The extensive body of work on feature selection could be also employed and, therefore, boost the accuracy of the overall inference.

SSR literature offers an arsenal of theoretical indicators and metrics that we showed correlate well with the performance as quantified by the precision and recall curves. Even though there is a growing research area for dynamical system inference algorithms, limited number of attempts to compute and exploit such metrics can be found in biological studies. The presented examples revealed that the values of these metrics could be easily lay far from the theoretically desirable. For instance, MIP took values closer to one rather than to zero in the mass cytometry example necessitating the design of additional experiments or the elimination of some problematic proteins from the dictionary. Actually, the determination of the dictionary is crucial in biological inverse problem inference. Quantities that are constant over time can imperil the accuracy of a structure learning algorithm because of the addition of collinearities especially when quadratic terms are considered in the dictionary. Thus, it is preferable to remove some or all of the constant-over-time variables from the dictionary and attempt to infer the structure of the quantities that are time-varying. Both incoherence metrics can serve as a guideline for the construction of a dictionary with high potential for true recovery.

Dynamics in biological processes contain critical information about the underlying reaction mechanisms between molecules. Current technologies are able to measure several time-points increasing the possibility of inducing the interactions between the measured quantities. However, the shape characteristics of the biological dynamics are usually simple. Prominent examples are impulsive patterns, which are either up-regulating or down-regulating excitations followed by a return to their basis values, and sustained patterns where the measured quantity remains over-expressed or under-expressed after the excitation (Bar-Joseph *et al.*, 2012). A cascade of four impulsive responses for protein signaling whose interactions were correctly inferred is shown in the upper plot of Figure 2a. Thus, the dynamical system that can be potentially identified correctly from relatively simple trajectories should not have complex driving forces. This is the primary reason why in our experiments we chose a linear dictionary. Adding more complex interactions to the dictionary will only result in less identifiable inference problems.

Finally, the presented examples assumed a linear with respect to the state variables dynamical model and, thus, a linear dictionary is constructed. However, the proposed inference algorithm is not restricted to linear differential equations. In Supplementary S6 Text, we apply the proposed dynamical inference algorithm to a non-linear and chaotic system from climate science, namely, Lorenz96 (Lorenz, 1996) where comparisons with SINDy are also performed. The precision-recall results indicate that both methods are able to achieve perfect reconstruction. However, SINDy requires 3–4 times less data in order to succeed it for the case of moderate chaotic behavior. Generally, chaotic systems enjoy richer and more complex dynamics which actually assist the structural learning of the differential equations as both the incoherence metrics and the obtained results on the accuracy revealed.

## 5 Conclusions

In this paper, we present the USDL algorithm, a generic and unified approach to solve the sparse dynamical structure inference problem from temporal data. It is based on the weak formulation of differential equations where the dimension of time is eliminated. Several properties of weak formulation such as being derivative free are useful and have high practical value. The transformed system is a set of linear equations whose sparse solution can be found using SSR algorithms. Convex relaxation methods as well as greedy algorithms such as OMP can be used and theoretical guarantees can be computed. To this end, a priori metrics such as MIP and a posteriori metrics such as ERC are computed and the satisfiability of the assumptions is checked. These metrics might also serve as candidates for optimization in experimental design. Moreover, various dynamical models can be transformed with the weak formulation to an SSR problem making our approach model independent. We test and compare USDL against SINDy on a wide range of dynamical models and show that, under high stochasticity, USDL achieves perfect reconstruction given enough data while SINDy fails for the same amount of data. This is notably evident for the stationary OU process where noise is prevalent revealing the generality and robustness of the proposed approach.

## Supplementary Material

btz065_Supplementary_Materials

## References

[btz065-B1] August E., Papachristodoulou A. (2009) Efficient, sparse biological network determination. BMC Syst. Biol., 3, 25. 19236711 10.1186/1752-0509-3-25 PMC2671484

[btz065-B2] Bar-Joseph Z. et al (2012) Studying and modelling dynamic biological processes using time-series gene expression data. Nat. Rev. Genet., 13, 552–564. 22805708 10.1038/nrg3244

[btz065-B3] Bartoszek K. et al (2012) A phylogenetic comparative method for studying multivariate adaptation. J. Theor. Biol., 314, 204–215. 22940235 10.1016/j.jtbi.2012.08.005

[btz065-B4] Bento J. et al (2010) Learning networks of stochastic differential equations. In: Lafferty J.D. et al (eds) Advances in Neural Information Processing Systems. Vol. 23. Curran Associates, Inc., Vancouver, British Columbia, Canada, pp. 172–180.

[btz065-B5] Bodenmiller B. et al (2012) Multiplexed mass cytometry profiling of cellular states perturbed by small-molecule regulators. Nat. Biotechnol., 30, 858–867. 22902532 10.1038/nbt.2317 PMC3627543

[btz065-B6] Bolstad A. et al (2011) Causal network inference via group sparse regularization. IEEE Trans. Signal Process., 59, 2628–2641. 21918591 10.1109/TSP.2011.2129515 PMC3170781

[btz065-B7] Bonneau R. et al (2006) The inferelator: an algorithm for learning parsimonious regulatory networks from systems-biology data sets de novo. Genome Biol., 7, R36. 16686963 10.1186/gb-2006-7-5-r36 PMC1779511

[btz065-B8] Bruckstein A.M. et al (2009) From sparse solutions of systems of equations to sparse modeling of signals and images. SIAM Rev., 51, 34–81.

[btz065-B9] Brunton S.L. et al (2016) Discovering governing equations from data by sparse identification of nonlinear dynamical systems. Proc. Natl. Acad. Sci. USA, 113, 3932–3937. 27035946 10.1073/pnas.1517384113 PMC4839439

[btz065-B10] Cai T.T., Wang L. (2011) Orthogonal matching pursuit for sparse signal recovery with noise. IEEE Trans. Inf. Theory, 57, 4680–4688.

[btz065-B11] Candes E., Tao T. (2005) Decoding by Linear Programming. IEEE Trans. Inf. Theory, 51, 4203–4215.

[btz065-B12] Candès E.J. et al (2006) Stable signal recovery from incomplete and inaccurate measurements. Commun. Pure Appl. Math., 59, 1207–1223.

[btz065-B13] Charbonnier C. et al (2010) Weighted-LASSO for structured network inference from time course data. Stat. Appl. Genet. Mol. Biol., 9. doi: 10.2202/1544-6115.1519. 10.2202/1544-6115.1519 20196750

[btz065-B14] Craven P., Wahba G. (1978) Smoothing noisy data with spline functions - Estimating the correct degree of smoothing by the method of generalized cross-validation. Numer. Math., 31, 377–403.

[btz065-B15] Daniels B.C., Nemenman I. (2015) Automated adaptive inference of phenomenological dynamical models. Nat. Commun., 6, 8. 10.1038/ncomms9133 PMC4560822 26293508

[btz065-B16] Davis G. et al (1997) Adaptive greedy approximations. Constr. Approx., 13, 57–98.

[btz065-B17] Davis M.E. (1984) Numerical Methods and Modeling for Chemical Engineers. John Wiley & Sons, Hoboken, New York, NJ.

[btz065-B18] DiStefano J.J. (2015) Dynamic Systems Biology Modeling and Simulation. Academic Press, Cambridge, MA.

[btz065-B19] Donoho D. (2006) Compressed sensing. IEEE Trans. Inf. Theory, 52, 1289–1306.

[btz065-B20] Donoho D., Huo X. (2001) Uncertainty principles and ideal atomic decomposition. IEEE Trans. Inf. Theory, 47, 2845–2862.

[btz065-B21] Evans L.C. (1998) Partial Differential Equations. Vol. 19. American Mathematical Society, Providence, RI.

[btz065-B22] Foucart S., Rauhut H. (2013) A Mathematical Introduction to Compressive Sensing. Applied and Numerical Harmonic Analysis. Springer, New York.

[btz065-B23] Friedman J. et al (2008) Sparse inverse covariance estimation with the graphical lasso. Biostatistics, 9, 432–441. 18079126 10.1093/biostatistics/kxm045 PMC3019769

[btz065-B24] Friston K.J. et al (2003) Dynamic causal modelling. NeuroImage, 19, 1273–1302. 12948688 10.1016/s1053-8119(03)00202-7

[btz065-B25] Gardiner C. (2004) Handbook of Stochastic Methods: For Physics, Chemistry & the Natural Sciences. Springer-Verlag, New York.

[btz065-B26] Gardiner C. (2009) Stochastic Methods: A Handbook for the Natural and Social Sciences. Springer, Berlin.

[btz065-B27] Gennemark P., Wedelin D. (2014) ODEion - a software module for structural identification of ordinary differential equations. J. Bioinform. Comput. Biol., 12, 1350015. 24467754 10.1142/S0219720013500157

[btz065-B28] Gustafsson M. et al (2009) Reverse engineering of gene networks with LASSO and nonlinear basis functions. Ann. N. Y. Acad. Sci., 1158, 265–275. 19348648 10.1111/j.1749-6632.2008.03764.x

[btz065-B29] Guyon I. et al (2003) An Introduction to Variable and Feature Selection. J. Mach. Learn. Res., 3, 1157–1182.

[btz065-B30] Kanehisa M., Goto S. (2000) KEGG: kyoto encyclopedia of genes and genomes. Nucleic Acids Res., 28, 27–30. 10592173 10.1093/nar/28.1.27 PMC102409

[btz065-B31] Klimovskaia A. et al (2016) Sparse regression based structure learning of stochastic reaction networks from single cell snapshot time series. PLOS Comput. Biol., 12, e1005234. 27923064 10.1371/journal.pcbi.1005234 PMC5140059

[btz065-B32] Krishnaswamy S. et al (2014) Conditional density-based analysis of T cell signaling in single-cell data. Science, 346, 1250689. 25342659 10.1126/science.1250689 PMC4334155

[btz065-B33] Lente G. (2015) Deterministic Kinetics in Chemistry and Systems Biology. SpringerBriefs in Molecular Science. Springer International Publishing, Basel, Switzerland.

[btz065-B34] Lorenz E.N. (1996) Predictability: a problem partly solved. In: Seminar on Predictability. Vol. 1, pp. 1–18. ECMWF, Shinfield Park, Reading.

[btz065-B35] Mallat S., Zhang Z. (1993) Matching pursuits with time-frequency dictionaries. IEEE Trans. Signal Process., 41, 3397–3415.

[btz065-B36] Mangan N.M. et al (2016) Inferring biological networks by sparse identification of nonlinear dynamics. IEEE Trans. Mol. Biol. Multi-Scale Commun., 2, 52–63.

[btz065-B37] Newman M.E.J. (2014) Networks: An Introduction. Oxford University, Oxford, pp. 163–186.

[btz065-B38] Oksendal B. (1985) Stochastic Differential Equations: An Introduction with Applications. Springer-Verlag, New York.

[btz065-B39] Papoutsoglou G. et al (2017) Scenery: a web application for (causal) network reconstruction from cytometry data. Nucleic Acids Res., 45, W270–W275. 28525568 10.1093/nar/gkx448 PMC5570263

[btz065-B40] Pati Y. et al (1993) Orthogonal matching pursuit: recursive function approximation with applications to wavelet decomposition. In: Proceedings of 27th Asilomar Conference on Signals, Systems and Computers, pp. 40–44. IEEE Computer Society Press, Pacific Grove, CA, USA.

[btz065-B41] Peifer M., Timmer J. (2007) Parameter estimation in ordinary differential equations for biochemical processes using the method of multiple shooting. IET Syst. Biol., 1, 78–88. 17441551 10.1049/iet-syb:20060067

[btz065-B42] Ramsay J.O. et al (2007) Parameter estimation for differential equations: a generalized smoothing approach. J. R. Stat. Soc. Ser. B Stat. Methodol., 69, 741–796.

[btz065-B43] Sachs K. et al (2005) Causal protein-signaling networks derived from multiparameter single-cell data. Science, 308, 523–529. 15845847 10.1126/science.1105809

[btz065-B44] Strang G., Fix G.J. (2008) An Analysis of the Finite Element Method. 2nd edn Wellesley-Cambridge Press, Wellesley, MA.

[btz065-B45] Tibshirani R. (1994) Regression shrinkage and selection via the lasso. J. R. Stat. Soc. Ser. B, 58, 267–288.

[btz065-B46] Tropp J. (2004) Greed is good: algorithmic results for sparse approximation. IEEE Trans. Inform. Theory, 50, 2231–2242.

[btz065-B47] Tropp J.A. (2006) Just relax: convex programming methods for identifying sparse signals in noise. IEEE Trans. Inform. Theory, 52, 1030–1051.

[btz065-B48] Tropp J.A., Gilbert A.C. (2007) Signal recovery from random measurements via orthogonal matching pursuit. IEEE Trans. Inform. Theory, 53, 4655–4666.

[btz065-B49] Zhan C., Yeung L.F. (2011) Parameter estimation in systems biology models using spline approximation. BMC Syst. Biol., 5, 14. 21255466 10.1186/1752-0509-5-14 PMC3750107

