# Supplementary material for: A unified approach for sparse dynamical system inference from temporal measurements
Source: Bioinformatics. 2018 Jan 31;35(18):3387–96. doi: 10.1093/bioinformatics/btz065 (PMC6748758; doi:10.1093/bioinformatics/btz065)
Supplement: btz065_Supplementary_Materials [file btz065_supplementary_materials.zip › btz065-suppl_data/SI1_Text.pdf]

Weak formulation for various differential equations

Yannis Pantazis and Ioannis Tsamardinos

The theory of the weak formulation has been developed in applied mathematics and particularly in the theory as well as in the numerical analysis of partial differential equations (PDEs). However, any dynamical system can be written in a weak form. This section presents the derivation of the weak formulation for ODEs, PDEs, stochastic differential equations (SDEs) and multivariate autoregressive models (MARs). Thus, a wide range of dynamical systems is covered. We also discuss various choices for the test functions. As we see in S5 Text, the choice of test functions can play a critical role in the perfect reconstruction of a dynamical system.

1 Ordinary differential equations

As presented in the main text, an ODE system with linear parameters is given in a non-matrix form by

$$\dot{x}_n = \sum_{q=1}^{Q} a_{nq}\psi_q(x) \ , \quad x_n(0) = x_{0n} \ , \quad n = 1, ..., N \ . \tag{1}$$

where $x_n(t)$ is the n-th state variable of the system while $\psi_q(x)$ is the q-th candidate function (or atom or element) of the dictionary.

In order to define the weak formulation, a set of test functions indexed by $m = 1, ..., M$ and denoted by $\phi_m(t)$ has to be specified. The number of test functions, M can be infinite but for practical purposes it is always finite. For the rest of the paper, we will assume that it is finite. Then, by multiplying (1) with $\phi_m(t)$ and integrating from 0 to T, we get for each n that

$$\int_0^T \dot{x}_n(t)\phi_m(t)dt = \int_0^T \sum_{q=1}^{Q} a_{nq}\psi_q(x(t))\phi_m(t)dt \ . \tag{2}$$

It is rewritten as

$$\langle \dot{x}_n, \phi_m \rangle = \sum_{q=1}^{Q} a_{nq} \langle \psi_q(x), \phi_m \rangle \ , \tag{3}$$

where $\langle f, g \rangle = \int_0^T f(t)g(t)dt$ is the inner product between two functions f and g in the L_2 function space. Notice that the infinite number of equations (one for each time instant $t \in [0, T]$) in (1) is transformed into M equations (one for each test function). The above set of equations is an atemporal system of equations whose matrix form is given in equation [2] of the main text.

Moreover, we assume that the test functions are smooth, having at least first derivative, hence, the differentiation of the state variables can be avoided using integration by parts. Indeed, it holds for each n that

$$x_n(t)\phi_m(t)\Big|_0^T - \langle x_n, \dot{\phi}_m \rangle = \sum_{q=1}^{Q} a_{nq} \langle \psi_q(x), \phi_m \rangle \ . \tag{4}$$

This is a key feature of weak formulation both theoretically and practically. From a practical point of view, there is no need to numerically estimate the time derivatives of the state variables. As discussed, differentiation in general amplifies the noise of a signal resulting in high variance estimates of the derivatives deteriorating the performance of any inference approach which necessitates derivative approximation. The weak formulation avoids differentiating the noisy state variables by exploiting the integration by parts theorem as shown above.

1.1 Test functions

The test functions utilized in weak formulation are smooth, not necessary orthogonal and can be chosen from a huge repository of functions. Examples are polynomials, Fourier/Laplace basis functions, B-splines, etc. Furthermore, the number of test functions should be minimum so as to reduce the computational complexity of the overall optimization problem. The choice of test functions could affect the performance of the inference procedure, hence, careful and educated

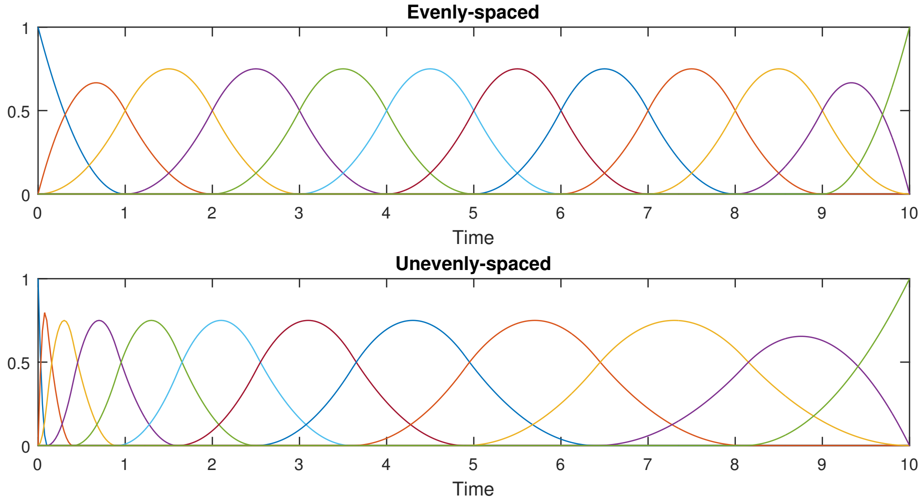

Figure 1: Upper plot: A set of 12 evenly-spaced B-splines in the interval $[0, 10]$. Lower plot: A set of 12 unevenly-spaced B-splines in the interval $[0, 10]$ where more resolution is put on the early times.

selection is needed for optimal performance. Of course, the optimal selection of test functions depends on the specific problem at hand and there is no family of functions that will perform optimally for all cases. The most suitable test functions for phenomena that has periodicities are Fourier modes. A set of M Fourier modes is defined by

$$\phi_1(t) = 1 \quad , \quad \phi_{2m-1}(t) = \cos\left(\frac{2\pi mt}{T}\right) \quad \text{and} \quad \phi_{2m}(t) = \sin\left(\frac{2\pi mt}{T}\right) \quad , \quad m = 1, ..., (M-1)/2 \tag{5}$$

with T being the final time. In this paper, Fourier modes are used as the default choice of test functions since they form a complete basis of functions in the L_2 function space. Another important class of test functions are the B-spline functions. B-splines with or without equally-spaced knots constitute another set of test functions that we explore in our demonstration examples. The advantage of using unequally-spaced knots is that specific areas of interest can be indicated and exploited. For instance in cellular protein signaling, if the dynamical phenomenon is stronger at the beginning then more knots will be assigned during the early times. Figure 1 demonstrates a set of 12 B-spline functions both with evenly-spaced knots (upper panel) and with unevenly-spaced knots (lower panel).

A third class of test functions are data-dependent test functions which can capture optimally the variations of the measured time-series. Similar to Karhunen-Loève expansion, spectral analysis of the measurement matrix reveals the principal components that explains the time-series. The principal components with the highest energy (i.e., eigenvalues) are used as test functions. The principal components are numerically computed through the use of singular value decomposition. We remark that this approach is known as principal component analysis or proper orthogonal decomposition.

2 Partial Differential Equations

PDEs constitute another class of dynamical systems where the variables depend not only on time but also on space. One of the simplest and well studied PDEs is heat equation which reads in 1D $x_t - x_{uu} = 0$ with some initial and boundary conditions. We denote with $x_t = \frac{\partial x}{\partial t}$ and $x_{uu} = \frac{\partial^2 x}{\partial u^2}$ the first order time-derivative and second-order space-derivative, respectively. For our purposes, we consider systems which are linear with respect to the unknown parameters. Restricting to one space dimension and one variable, the equation can be of the general form

$$\sum_{q=1}^{Q} a_q \psi_q(x, x_t, x_u, x_{tt}, x_{tu}, x_{uu}, ...) = 0 \tag{6}$$

where the driving functions, $\{\psi_q\}$, depend in the physical or engineering application. Several equations fall in the above general form. To name a few, transport equation (first order), heat equation, Laplace's equation (both parabolic PDEs) and wave equations with or without shocks, with or without interactions (hyperbolic PDEs). Additionally, special but general cases from reaction-diffusion equations (Fisher-Kolmogorov equation), chemotaxis, master equation as well as Fokker-Planck equation (with the last two belonging to the class of Kolmogorov equations) can be written in the general form given by (6).

Typically, the inference (or inverse) problems in PDEs try to estimate the boundaries or some other parameters of the PDE and not the driving forces that governs the system since the kinetic laws are assumed known. Nevertheless, in this paper, we want to show the generality of the weak formulation approach to transform a PDE into an atemporal system of equations. Interestingly, the weak formulation is best applied to finding the solution of PDEs. Proceeding, the test functions are now both time and space dependent and the integration is performed in both dimensions. So, let $\{\phi_m(t,x)\}_{m=1}^M$ be a set of M test functions, then, the weak form of (6) is

$$\sum_{q=1}^{Q} a_{nq}\langle\psi_q(x,x_t,x_u,x_{tt},x_{tu},x_{uu},...),\phi_m\rangle = 0 \tag{7}$$

where $\langle f,g\rangle = \int_{\mathcal{D}}\int_0^T f(t,u)g(t,u)dtdu$ is the inner product adapted for functions with both time and space arguments. $\mathcal{D}$ is the space domain of the free variable. Notice that the integration by parts trick can be applied for both time-derivatives and space-derivatives. Thus, the need for numerical estimation of the derivatives is minimized. Moreover, a standard choice for time-space test functions is to assume that time and space are separated. Indeed, the test functions are defined as $\phi_m(t,u) = \phi_{m,1}(t)\phi_{m,2}(u)$ where $\phi_{m,1}$ and $\phi_{m,2}$ are test functions as in the previous subsection.

Finally, we would like to notice that our primary intention is to show that weak formulation is also applicable to PDEs without any difficulty in the derivation of the atemporal system. However, the study of particular PDEs may necessitate special treatment especially on the sampling scheme of the solution as well as on the suitable selection of test functions. An extensive discussion on PDE dynamical system inference is beyond the scope of this publication.

3 Stochastic Differential Equations

A stochastic process $x(t,\omega)$ is a function of two variables, time t and random outcome ω. For a fixed random variable ω, it is a function of time, $x(t) := x(t,\omega)$ which is called a realization, time-series or trajectory of the process. In the following, we suppress the dependence on the random element and keep only the dependence on time. Proceeding, consider an N-dimensional stochastic process, $x(t)$ which is driven by an SDE with additive noise

$$\dot{x}_n = \sum_{q=1}^{Q} a_{nq}\psi_q(x) + \sigma\dot{B}_n\ , \quad n = 1,...,N, \tag{8}$$

where $B_n(t)$ are independent Brownian motions for each $n = 1,...,N$. Brownian motion as a function of time is nowhere differentiable thus its time derivative can be rigorously defined only through the weak formulation. However, and, in an intuitive level, the time derivative of a Brownian motion is known as the white noise, and, it is also part of the driving forces of this stochastic dynamical system. The more standard notation for SDEs is based on differentials and it is given by the following formula

$$dx_n(t) = \sum_{q=1}^{Q} a_{nq}\psi_q(x(t))dt + \sigma dB_n(t)\ , \quad n = 1,...,N. \tag{9}$$

As before the weak formulation is obtained by multiplication of the test function and integration. Thus, for $m = 1,...,M$, we get

$$\int_0^T \phi_m(t)dx_n(t) = \sum_{q=1}^{Q} a_{nq}\int_0^T \phi_m(t)\psi_q(x(t))dt + \sigma\int_0^T \phi_m(t)dB_n(t)\ , \quad n = 1,...,N\ . \tag{10}$$

However there is an important difference between the standard Riemann integration used in ODEs and PDEs and stochastic integrals. The interpretation of the integrals is different which results in different approaches when numerical estimation is performed. Here, the stochastic integrals are Ito integrals [1] which are defined in a similar manner to the Riemann-Stieltjes integrals. Numerically, Ito integral is approximated by the Riemann sum

$$\int_0^T f(t)dx_t \approx \sum_{i=0}^{K-1} f(t_i)\big(x(t_{i+1}) - x(t_i)\big) \tag{11}$$

where $\{t_i\}_{i=0}^K$ denotes a partition of the interval $[0,T]$. Notice that there exist a variant of the integration by parts theorem which holds for Ito integrals. However, we did not utilize it in the demonstrated SDE example.

4 Multivariate Autoregressive Model

The weak formulation can be also applied on discrete-time dynamical systems. The approach is similar to the continuous time except the definition of inner products where the integration is replaced by summation. We demonstrate the weak

form of discrete-time dynamical systems with multivariate autoregressive (MAR) model which additionally is stochastic. In MAR models, the current measurement is a linear combination of the previous measurements plus a stochastic white noise (i.e., Gaussian and independent). The mathematical formula for MAR model is

$$x(t) = \sum_{\tau=1}^{\mathcal{T}} A(\tau)x(t-\tau) + e(t) \ , \quad t = 0, 1, ..., T \tag{12}$$

where $x(t) \in \mathbb{R}^N$ is the discrete-time N-dimensional MAR process, $A(\tau)$ is the connectivity matrix for the τ-th previous measurement while $e(t)$ is the driving noise term. $\mathcal{T}$ determines the order of MAR.

The weak formulation for a discrete-time system is defined for a set of test functions $\{\phi_m\}_{m=1}^{M}$ whose domain is the set of natural numbers. The new atemporal system is given by

$$\langle x_n, \phi_m \rangle = \sum_{\tau=1}^{\mathcal{T}} \sum_{q=1}^{Q} a_{nq}(\tau) \langle x_q(t-\tau), \phi_m \rangle + \langle e_n, \phi_m \rangle \tag{13}$$

where the inner product is now defined as $\langle f, g \rangle = \sum_{t=0}^{T} f(t)g(t)$. Defining the cross-correlation function between two functions as $C_{fg}(\tau) = \sum_{t=0}^{T} f(t)g(t+\tau)$, the system of weak-form equations becomes

$$C_{x_n \phi_m}(0) = \sum_{\tau=1}^{\mathcal{T}} \sum_{q=1}^{Q} a_{nq}(\tau) C_{x_q \phi_m}(-\tau) + C_{e_n \phi_m}(0) \ , \tag{14}$$

which is a linear system of equations. Test functions can be discrete versions of Fourier modes or B-spline functions. Using as test functions, the Dirac delta function (i.e., $\phi_m(t) = \delta(t-m)$) then the weak formulation falls back into the "temporal" formulation studied in [2].

References

[1] Thomas Mikosch. *Elementary stochastic calculus with finance in view.* World Scientific, 2003.

[2] Andrew Bolstad, Barry D. Van Veen, and Robert Nowak. Causal network inference via group sparse regularization. *IEEE Transactions on Signal Processing*, 59(6):2628–2641, 2011.
