# Supplementary material for: A unified approach for sparse dynamical system inference from temporal measurements
Source: Bioinformatics. 2018 Jan 31;35(18):3387–96. doi: 10.1093/bioinformatics/btz065 (PMC6748758; doi:10.1093/bioinformatics/btz065)
Supplement: btz065_Supplementary_Materials [file btz065_supplementary_materials.zip › btz065-suppl_data/SI2_Text.pdf]

Time-series Data Fitting a.k.a. Collocation Method

Yannis Pantazis and Ioannis Tsamardinos

A sample of a time-course dataset (i.e., a non-repeated measurement) is denoted as

$$y_{nkp} = y_n^{(p)}(t_k) \tag{1}$$

where $n = 1, ..., N$ with N being the number of species, $k = 1, ..., K$ with K being the total number of sampling points while $p = 1, ..., P$ with P being the number of measurements. The interpretation is that y_{nkp} is the p-th measurement of the n-th species at time instant, t_k. We set $t_0 = 0$ and $t_K = T$ be the initial and final time points, respectively. Notice that the number of measurements may depend on the time index k (i.e., $P = P(k)$) but for simplicity reasons we assume that it is constant. It is also useful to define the P-dimensional measurement vector, y_n, as

$$y_n = [y_n^{(1)}(t_1), ..., y_n^{(1)}(t_K), ..., y_n^{(P)}(t_1), ..., y_n^{(P)}(t_K)]^T \tag{2}$$

The weak formulation does not apply directly when time-course data are provided. Thus, time-series interpolation is utilized. This is achieved by assuming that the state variables over time can be approximated by a linear combination of L basis functions of time, denoted by $\{\bar{\phi}_l(t)\}_{l=1}^L$. Thus, the n-th state variable is written as

$$x_n(t) = \sum_{l=1}^{L} c_{nl}\bar{\phi}_l(t) = c_n^T \bar{\phi}(t) \ , \quad n = 1, ..., N \ , \tag{3}$$

where $\bar{\phi}(t) = [\bar{\phi}_1(t), ..., \bar{\phi}_L(t)]^T \in \mathbb{R}^L$ is an L-dimensional vector whose elements are the basis functions. Typical choice for the basis functions are B-splines which are local in time and are defined by two parameters; the degree of the interpolating polynomials and the set of knots. Knots in B-spline construction are usually dictated by the sampling time points, $t_0, ..., t_K$, but they can be enriched or reduced in a user-specific basis. If necessary, they can be also optimized through a cross-validation procedure.

In order to formulate the data fitting optimization problem, define the $L \times (K+1)P$ matrix, $\tilde{\phi}$, as

$$\tilde{\phi} = \begin{bmatrix} \bar{\phi}_1(t_0) & ... & \bar{\phi}_1(t_0) & ... & \bar{\phi}_1(t_K) & ... & \bar{\phi}_1(t_K) \\ \vdots & & \vdots & & \vdots & & \vdots \\ \bar{\phi}_L(t_0) & ... & \bar{\phi}_L(t_0) & ... & \bar{\phi}_L(t_K) & ... & \bar{\phi}_L(t_K) \end{bmatrix} \ , \tag{4}$$

which is in alignment with the measurement vectors y_n in (2). The (penalized) data fitting problem is defined as

$$\min_C \sum_{n=1}^{N} \{||y_n - c_n^T \tilde{\phi}||_2^2 + \lambda_C c_n^T \ddot{\bar{\Phi}} c_n\} \tag{5}$$

where C is the $N \times L$ coefficient or weight matrix defined by

$$C = [c_1, ..., c_N]^T \ , \tag{6}$$

while the second term is a smoothing penalty controlled by the non-negative parameter (i.e., weight) λ_C. The second term is designed to penalize the ripples of the interpolated time-series by penalizing the squared L_2 norm of the derivatives of the time-series. This reads to $\int_0^T (\dot{x}_n(t))^2 dt = \langle \dot{x}_n, \dot{x}_n \rangle$ which can be rewritten as $c_n^T \ddot{\bar{\Phi}} c_j$ where $\ddot{\bar{\Phi}}$ is a $L \times L$ symmetric matrix with elements $\ddot{\bar{\Phi}}_{l,k} = \langle \dot{\bar{\phi}}_l, \dot{\bar{\phi}}_k \rangle$. The derivation of the ripple-penalty is

$$\begin{aligned} \int_0^T (\dot{x}_n(t))^2 dt &= \int_0^T \big(\sum_{l=1}^{L} c_{nl}\dot{\bar{\phi}}_l(t)\big)\big(\sum_{k=1}^{L} c_{nk}\dot{\bar{\phi}}_k(t)\big) dt \\ &= \sum_{l=1}^{L}\sum_{k=1}^{L} c_{nl}c_{nk}\langle \dot{\bar{\phi}}_l, \dot{\bar{\phi}}_k \rangle = c_n^T \ddot{\bar{\Phi}} c_n \end{aligned}$$

The optimization problem in (5) is a regularized Least Squares (RLS) problem. Additionally, it can be decoupled into N independent optimization subproblems; one for each column of C. Thus, the solution for the n-th column (i.e., the n-th coefficient vector) is given by

$$\hat{c}_n = \left(\tilde{\phi}\tilde{\phi}^T + \lambda_C \ddot{\Phi}\right)^{-1} \tilde{\phi} y_n^T \ , \tag{7}$$

with $n = 1, ..., N$.

Cost functional (5) can be seen from a *Bayesian perspective* as a sum of the log-likelihood and the log-prior distribution. The assumption incorporated in the prior distribution is that the trajectories are typically smooth functions of time. Both likelihood and prior distributions are Gaussian thus the data fitting problem is a linear optimization problem for the coefficient matrices which can be solved explicitly as shown above. We would like to highlight also that a simple approach to create many trajectories is to repeatedly and randomly choose a subset of the measurements and solve (5). A weight can be also assigned to each trajectory from the posterior distribution defined by (5). The expectation when one feeds several trajectories to the learning algorithm is that the dynamics of the underlying process are more accurately sampled, thus, the performance is improved. Finally, there are cases where the distribution of the measurements are multi-modal or heteroscedastic. In such cases, the Gaussianity assumption is not valid resulting in sub-optimal solutions. Hence the cost functional should be enriched with multi-modal distributions such as the non-linear Gaussian Mixture Model. Alternatively, Monte Carlo methods which are more expensive computationally can be utilized for the sampling of the trajectories in a non-Gaussian case.
