# Supplementary material for: A unified approach for sparse dynamical system inference from temporal measurements
Source: Bioinformatics. 2018 Jan 31;35(18):3387–96. doi: 10.1093/bioinformatics/btz065 (PMC6748758; doi:10.1093/bioinformatics/btz065)
Supplement: btz065_Supplementary_Materials [file btz065_supplementary_materials.zip › btz065-suppl_data/SI3_Text.pdf]

Tackling Multiple Interventions

Yannis Pantazis and Ioannis Tsamardinos

Depending on the application at hand, there are various types of interventions that can be applied. Probably, the simplest intervention is to start from different initial values, then let the system evolve and measure it again. The wider exploration of the state space results in more informative measurements thus the estimation of the structure and the parameters of the dynamical system becomes in principle more identifiable. In systems biology for instance, such type of interventions are called activations. Another type of intervention is (partial) inhibition where the effect of a state variable is neutralized or kept constant over time. For instance, if n^* state variable is kept constant then we can impose the constraint $x_{n^*}(t) = c_0$ for all t or equivalently $\dot{x}_{n^*}(t) = 0$ which results to the constraint

$$\sum_{q=1}^{Q} a_{n^*q}\psi_q(x) = 0 \ .$$

When an ODE system is augmented with constraints that do not contain time derivatives then the resulted set of equations is called a system of differential algebraic equations. More complex interventions and/or physical constraints (or laws) can be incorporated into the ODE system. The intervention type where $c_0 = 0$ is typically called inhibition.

However, experimentalists and scientists do not have full control on the effect of an intervention thus it is preferable to introduce a general way to model interventions other than hard constraints. Indeed, for ODE systems, the original equation, $\dot{x}_n = \sum_{q=1}^{Q} a_{nq}\psi_q(x)$, can be slightly extended to

$$\dot{x}_n = \sum_{q=1}^{Q} a_{nq}\psi_q(x) + b_n u_n \ , \quad x_n(0) = x_{0n} \ , \quad n = 1, ..., N \ , \tag{1}$$

where $b_n \in \mathbb{R}$ while $u_n = u_n(t)$ is a given function of time which can be thought as an input signal and constitutes the intervention signal. If for instance n^* state variable is inhibited then this intervention is approximated by the reaction $X_{n^*} \to \emptyset$ with a large rate constant. In the modified ODE system (1), intervention of variable n^* is modeled by setting $u_n = 0$ for $n \neq n^*$ and $u_{n^*} = x_{n^*}$. Moreover, input signals can amount not only for inhibition but also for other type of interventions such as dosage level. Indeed, the form of the input signal for dosage intervention can be defined as $u_n(t) = u(t) = e^{-t/\tau}$ where τ controls the decay rate of the drug while b_n reflects the strength of the effect of the drug to the n-th state variable.

Proceeding, assume that there are R different experimental conditions thus there are R different ODE systems with each one having different intervention input $u_n^{(r)}$, $r = 1, ..., R$. The weak formulation for the r-th ODE and the n-th variable is given by

$$z_n^{(r)} = \Psi^{(r)} a_n + b_n^{(r)} v_n^{(r)} \tag{2}$$

where $z_n^{(r)}$ and $\Psi^{(r)}$ as in the main text while $v_n^{(r)}$ is the projection vector of the intervention input to the test functions with elements given by $v_{nm}^{(r)} = \langle u_n^{(r)}, \phi_m \rangle$. The integrated ODE system can be written in a matrix form as

$$\begin{bmatrix} z^{(1)} \\ \vdots \\ z^{(R)} \end{bmatrix} = \begin{bmatrix} \Psi^{(1)} & v^{(1)} & 0 & \cdots & 0 \\ \Psi^{(2)} & 0 & v^{(2)} & \cdots & 0 \\ \vdots & \vdots & \vdots & \vdots & \vdots \\ \Psi^{(R)} & 0 & 0 & \cdots & v^{(R)} \end{bmatrix} \begin{bmatrix} a \\ b^{(1)} \\ \vdots \\ b^{(R)} \end{bmatrix} \tag{3}$$

The derived system of equations fall again in the SSR category thus OMP can be utilized. Moreover, since we know a priori that there is driving input we start OMP with index set $\mathcal{S} = \{Q+1, \cdots, Q+R\}$. Finally, we remark that when $v^{(r)} = 0$ for some r then there is no input term. Hence, it becomes redundant in the above system of equations and the corresponding column of the measurement matrix as well the corresponding $b^{(r)}$ coefficient are removed.
