# Supplementary material for: A unified approach for sparse dynamical system inference from temporal measurements
Source: Bioinformatics. 2018 Jan 31;35(18):3387–96. doi: 10.1093/bioinformatics/btz065 (PMC6748758; doi:10.1093/bioinformatics/btz065)
Supplement: btz065_Supplementary_Materials [file btz065_supplementary_materials.zip › btz065-suppl_data/SI4_Text.pdf]

Sparse Signal Recovery

Yannis Pantazis and Ioannis Tsamardinos

After the application of the weak formulation, the transformed system of equations is written as

$$z = \Psi a + e \tag{1}$$

where z, Ψ and a as defined in the main text while e denotes the noise vector with $||e||_2 = \epsilon$ being the energy of the error/noise term. The SSR problem is defined as an l_0 quasi-norm minimization program

$$\min_a ||a||_0 \text{ subject to } ||z - \Psi a||_2 \leq \epsilon \ , \tag{l0-error}$$

where l_0 quasi-norm counts the number of non-zero elements in its argument (i.e., $||a||_0 = \#\{q : a_q \neq 0\}$). In order to solve this non-convex program, one must search through all possible solutions making this approach intractable for large systems since the search space is exponentially large [1]. There is a wide spectrum of algorithms that try to approximate the solution of the above intractable program. We refer to [2] for a recent review. In the following, we present representative approximation algorithms for two different families that solve SSR. Moreover, the presented techniques are accompanied with theoretical guarantees which determine under which conditions the approximate solution is also the solution of (l0-error).

Let us remark here that the columns of the measurement matrix are assumed to be normalized in the literature, however, Ψ obtained by weak formulation has not normalized columns. In order to make the columns of Ψ having l_2 norm equal to 1, the following computation is performed,

$$z = \Psi a + e = \sum_{q=1}^{Q} a_q \psi_q + e = \sum_{q=1}^{Q} a_q ||\psi_q||_2 \frac{\psi_q}{||\psi_q||_2} + e = \sum_{q=1}^{Q} \bar{a}_q \bar{\psi}_q + e = \bar{\Psi}\bar{a} + e \ . \tag{2}$$

Thus, the non-zero coefficients of a are amplified by a factor which is proportional to the l_2-norm of the respective column of Ψ.

1 Convex Relaxation Theory

One approach to relax the above optimization problem and make it computationally feasible is to replace the l_0 quasi-norm with the l_1 norm and obtain the convex program

$$\min_a ||a||_1 \text{ subject to } ||z - \Psi a||_2 \leq \delta \ , \tag{l1-error}$$

where $||a||_1 = \sum_{q=1}^{Q} |a_q|$ is the l_1 norm while δ is the tolerance. It holds that l_1 norm is the closest convex function to l_0 quasi-norm [3]. Using standard linear programming software the (l1-error) minimization problem can be solved in polynomial time. Nevertheless, the solution of (l1-error) is just an approximation to the SSR problem and it is not guaranteed that it provides the same answer as the l_0 quasi-norm problem. Systematic investigations have been presented throughout the last fifteen years exploring when the solutions of the two problems are equal. For the sake of completeness, we provide a special case of a theorem by Tropp [3] which is based on ERC and determines under which conditions the solution of (l1-error) recovers the true support. In order to present the theorem, we have to define the following quantities. The (p, q)-matrix operator norm is defined for a matrix B as

$$||B||_{p,q} := \max_{x \neq 0} \frac{||Bx||_q}{||x||_p} \ ,$$

while, for an index set $\mathcal{S}$, we define the restricted pseudo-inverse operator over $\mathcal{S}$ as

$$\bar{\Psi}_{\mathcal{S}}^{\dagger} := (\bar{\Psi}_{\mathcal{S}}^T \bar{\Psi}_{\mathcal{S}})^{-1} \bar{\Psi}_{\mathcal{S}}^T \ ,$$

and the best l_2 approximation of z restricted on $\mathcal{S}$ as

$$\hat{z}_{\mathcal{S}} := \bar{\Psi}_{\mathcal{S}} \bar{\Psi}_{\mathcal{S}}^{\dagger} z \ .$$

Then, the following holds.

Theorem 1: (Tropp '06) *Let $\mathcal{T}$ be the support of the true solution for which $ERC(\mathcal{T}) \geq 0$ and select tolerance to be*

$$\delta = \sqrt{\epsilon^2 + \left(\frac{||\bar{\Psi}(z - \hat{z}_{\mathcal{T}})||_\infty ||\bar{\Psi}_{\mathcal{T}}^\dagger||_{2,1}}{ERC(\mathcal{T})} \right)^2} \ .$$

If for all $q \in \mathcal{T}$ holds that

$$|a_q| > \frac{||\bar{\Psi}_{\mathcal{T}}^\dagger||_{2,2}}{||\psi_q||_2} \delta$$

then the support of the (unique) solution of (l1-error) convex program with tolerance δ is exactly $\mathcal{T}$.

Another methodology to relax the l_0 quasi-norm optimization program is to consider the Lagrangian version of (l1-error). It reads

$$\min_a \frac{1}{2}||z - \Psi a||_2^2 + \lambda ||a||_1 \ , \qquad \text{(l1-penalty)}$$

where λ is a weight parameter that balances between complexity and sparsity. Indeed, increasing λ results in sparser solutions. This optimization program is also known as LASSO [4] and there exist fast algorithms such as LARS [5] which finds LASSO solutions. As in the previous case, there are theoretical guarantees under which conditions the LASSO solution is also the solution of the SSR problem. We provide two theorems; one involving MIP and the other ERC which are special cases of theorems in [3].

Theorem 2: (Tropp '06) *Let $\mathcal{T}$ be the support of the true solution and $k := |\mathcal{T}|$ its size. Suppose that $\mu \leq \frac{1}{2k}$ as well as that*

$$||\bar{\Psi}_{\mathcal{T}}(z - \hat{z}_{\mathcal{T}})||_\infty \leq \lambda \frac{1 - (2k-1)\mu}{1 - (k-1)\mu}$$

for some $\lambda > 0$ where $\hat{z}_{\mathcal{T}}$ is the best l_2 approximation over $\mathcal{T}$. If for all $q \in \mathcal{T}$ holds that

$$|a_q| > \frac{\lambda}{||\psi_q||_2 (1 - (k-1)\mu)}$$

then the support of the (unique) solution of the Lasso convex program with parameter λ is exactly $\mathcal{T}$.

It is noteworthy that for the noiseless case and given that $\mu \leq \frac{1}{2k}$, there is always a small enough λ which satisfies the condition on the non-zero coefficients of a. Moving forward, as we saw in the main text, the condition for MIP is rarely satisfied, mainly, due to collinearity between columns of Ψ that might be irrelevant to the space spanned by the atoms of the actual solution. For this reason, we stated that MIP is rather conservative. The following theorem, again due to Tropp, is based on ERC and determines when Lasso solution recovers the true support.

Theorem 3: (Tropp '06) *Let $\mathcal{T}$ be the support of the true solution for which $ERC(\mathcal{T}) \geq 0$. Suppose that*

$$||\bar{\Psi}_{\mathcal{T}}(z - \hat{z}_{\mathcal{T}})||_\infty \leq \lambda ERC(\mathcal{T})$$

for some $\lambda > 0$. If for all $q \in \mathcal{T}$ holds that

$$|a_q| > \frac{\lambda ||(\bar{\Psi}_{\mathcal{T}}^T \bar{\Psi}_{\mathcal{T}})^{-1}||_{\infty,\infty}}{||\psi_q||_2}$$

then the support of the (unique) solution of the Lasso convex program with parameter λ is exactly $\mathcal{T}$.

The condition on ERC is harder to validate compared to MIP because the support of the true but unknown solution is required. Here we present the results when the true support is known, however, in Tropp [3], the conditions are relaxed for arbitrary index set of linearly independent collection of dictionary atoms. Finally, we remark that a difficulty in Lasso formulation is that little or no information about the hyper-parameter λ is provided and scientists usually apply information criteria in order to select λ. Nevertheless, guidance on the values of λ can be provided from the above theorems since two counterbalancing conditions for λ need to be satisfied.

2 Orthogonal Matching Pursuit

There are several greedy algorithms proposed in the literature trying to solve the SSR problem. Among them one of the fastest and surely the most popular is OMP. Apart from being very fast, OMP has the important property that its hyper-parameter is easy to interpret and approximate from the available data. For the sake of completeness, we present the basic OMP algorithm where we added an extra criterion on the maximum allowed number of non-zero elements which is denoted with K.

- OMP Algorithm:

  1. Initialize the residual $r_0 = z$ and the set of selected indices $S = \emptyset$. Set counter to $i = 1$.
  2. Find the next index as
  $$q' = \arg\max_q |\bar{\psi}_q^T r_{i-1}|$$
  and add q' to S.
  3. Solve the Least Squares problem using only the selected indices
  $$\hat{a}_S = (\Psi_S^T \Psi_S)^{-1} \Psi_S^T z = \Psi_S^\dagger z$$
  Update residual error $r_i = z - \Psi_S \hat{a}_S$.
  4. If $||r_i||_2 < \epsilon$ or $|S| > K$, stop the algorithm. Otherwise, set $i = i + 1$ and return to step 2.

The stopping criterion in OMP requires the knowledge of the l_2 norm of the true residual error which in principle is not available a priori. The energy of the true residual error is approximated as

$$\hat{\epsilon} = (1 + \alpha)||r_{LS}||_2 \ ,$$

where r_{LS} is the residual when the complete dictionary is utilized given by $r_{LS} := z - \Psi\Psi^\dagger z$ while α is a small positive number in the range $10^{-1} - 10^{-3}$. α is user-defined and corresponds to the potential over-fitting of the complete model representation. Notice that the stopping criterion can be rewritten as

$$\frac{||r_i||_2 - ||r_{LS}||_2}{||r_{LS}||_2} < \alpha \ ,$$

implying that OMP stops when the relative residual energy becomes smaller than α.

In the following, we present two theorems on perfect support recovery; one based on MIP and the other on ERC which were first proven in [6].

Theorem 4: (Cai & Wang '11) *Suppose* $||e||_2 \leq \epsilon$ *and* $\mu < \frac{1}{2k-1}$ *where* k *is the number of nonzero elements of* a*. Then, OMP algorithm with stopping rule* $||r_i||_2 \leq \epsilon$ *recovers the true subset of correct dictionary atoms indexed by* $\mathcal{T}$ *if for all* $q \in \mathcal{T}$ *holds that*

$$|a_q| \geq \frac{2}{SNR(q)(1 - (2k - 1)\mu)} \ .$$

The next theorem collects the pieces that has been presented in the main text.

Theorem 5: (Cai & Wang '11) *Suppose* $||e||_2 \leq \epsilon$ *and* $ERC(\mathcal{T}) > 0$ *where* $\mathcal{T}$ *is the support of the true solution. Then, OMP algorithm with stopping rule* $||r_i||_2 \leq \epsilon$ *recovers the true subset of correct dictionary atoms,* $\mathcal{T}$*, if for all* $q \in \mathcal{T}$ *holds that*

$$|a_q| \geq \frac{2}{SNR(q)ERC(\mathcal{T})\lambda_{\min}(\mathcal{T})} \ .$$

Additional theoretical results can be found in [6]. For instance, by varying the stopping criterion, one can guarantee that the strongest components of the solution will be obtained showing that the strongest components are selected first.

2.1 Adding prior Knowledge

In some applications, user may partially know the driving forces of a dynamical system or she has an instrument that intervene the system in a systematic and known way. Thus, being able to add prior knowledge to the inference algorithm is very important. Another convenient feature of OMP is that adding prior knowledge is straightforward. Indeed, instead of starting with an empty index set, S in step 1 of OMP will be initialized with the provided indices that encode the prior knowledge. Accordingly, the initial residual error will be evaluated after the subtraction of the known non-zero components.

References

[1] B. K. Natarajan. Sparse Approximate Solutions to Linear Systems. *SIAM Journal on Computing*, 24(2):227–234, apr 1995.

[2] Zheng Zhang, Yong Xu, Jian Yang, Xuelong Li, and David Zhang. A Survey of Sparse Representation: Algorithms and Applications. *IEEE Access*, 3:490–530, 2015.

[3] Joel A. Tropp. Just relax: convex programming methods for identifying sparse signals in noise. *Information Theory, IEEE Transactions on*, 52(3):1030–1051, 2006.

[4] Robert Tibshirani. Regression selection and shrinkage via the lasso. *Journal of the Royal Statistical Society B*, 58(1):267–288, 1996.

[5] B. Efron, T. Hastie, I. Johnstone, and R. Tibshirani. Least angle regression. *Annals of Statistics*, 32(2):407–499, 2004.

[6] T. Tony Cai and Lie Wang. Orthogonal matching pursuit for sparse signal recovery with noise. *IEEE Transactions on Information Theory*, 57(7):4680–4688, 2011.
