# Supplementary material for: A unified approach for sparse dynamical system inference from temporal measurements
Source: Bioinformatics. 2018 Jan 31;35(18):3387–96. doi: 10.1093/bioinformatics/btz065 (PMC6748758; doi:10.1093/bioinformatics/btz065)
Supplement: btz065_Supplementary_Materials [file btz065_supplementary_materials.zip › btz065-suppl_data/SI5_Text.pdf]

Further Demonstrations

Yannis Pantazis and Ioannis Tsamardinos

We provide here several details on the production of the figures in the main text. Additional experiments are also presented for each of the demonstration examples.

1 Protein interaction network

This demonstration example emulates the output of a single-cell mass cytometry machine with a prototypical protein interaction network where protein P_1 interacts with protein P_3 through protein P_2. The complete biochemical reaction network is given in Table 1 and the corresponding ODE system has been simulated with standard ODE solver. It consists of six reactions which follow the law of mass action kinetics. R_1 and R_2 produce P_2 and P_3, respectively, while R_3 and R_4 correspond to the binding and unbinding of the P_3 to P_1. R_5 and R_6 are simple degradation mechanics. The initial concentration of the state variables (i.e., the species) are 1 for P_1 and 0 for the rest species. Furthermore, we perform four interventions. The first intervention was to change the initial concentration of P_2 from 0 to 5 while the second intervention was to change the initial concentration of P_3 from 0 to 15. The third intervention was the inhibition of P_2 while the final one was the inhibition of P_3. Inhibition was implemented by augmenting a new and fast degradation reaction with rate constant equal to 100.

Table 1: The reaction table with $P_1 - P_3$ corresponding to the 3 proteins while $[P_1P_3]$ corresponds to the Protein-Protein complex. The state of the reaction model is defined as $x = [P_1, P_2, P_3, [P_1P_3]]^T$.

Event	Reaction	Rate	Rate constant
R_1	$P_1 \to P_1 + P_2$	$a_1(x) = k_1 P_1$	$k_1 = 4$
R_2	$P_2 \to P_2 + P_3$	$a_2(x) = k_2 P_2$	$k_2 = 0.05$
R_3	$P_1 + P_3 \to [P_1P_3]$	$a_3(x) = k_b P_1 \cdot P_3$	$k_b = 0.01$
R_4	$[P_1P_3] \to P_1 + P_3$	$a_4(x) = k_u [P_1P_3]$	$k_u = 0.001$
R_5	$P_2 \to \emptyset$	$a_5(x) = k_{-2} P_2$	$k_{-2} = 0.4$
R_6	$P_3 \to \emptyset$	$a_6(x) = k_{-3} P_3$	$k_{-3} = 0.2$

The resulted dynamical system has a subtle property. Due to the fact that $dP_1/dt + d[P_1P_3]/dt = 0$, the following conservation law holds

$$P_1 + [P_1P_3] = 1 \ .$$

Eventually, the dynamical system consists of three equations with four unknowns. Therefore, the complete reaction network is inherently unidentifiable when the conservation law is not taken into account making it an inappropriate example for testing the USDL algorithm. This is the main reason why we choose a coarser and thus simpler dynamical model for the learning algorithms. Moreover, the property of unidentifiability is very typical in biochemical reaction networks and in conjunction with the sloppiness of the parameters [1], the structure inference of the complete biochemical network of interactions is impossible most of the times.

Figure 1(a) presents the F1 score (i.e., the harmonic mean between precision and recall) for both USDL (left column) and SINDy (right column) and various number of experiments and noise levels. Perfect reconstruction which is implied by an F1 score at 100% is achieved only by the USDL algorithm when the noise has low variance and data from all five experiments are used. As already stated in the main text, we choose the hyperparameter value that maximizes the F1 score. We also observe that utilizing up to $R = 3$ experiments results has similar F1 score performance for both approaches however the USDL algorithm is superior when all available data are exploited.

Figure 1(b) presents the time-series forecasts (black curves) based on the estimated parameter values for both USDL (left column) and SINDy (right column). Our simulation is performed on a new experiment which is different from the five experiments used for the inference. Indeed, the initial concentration is 10 for P_2 and 5 for P_3. Let us remark also that we use as initial values to the ode solver the mean of variable's concentration at time point 0. In accordance with the ERC metric, the problematic variable with the highest error is P_2.

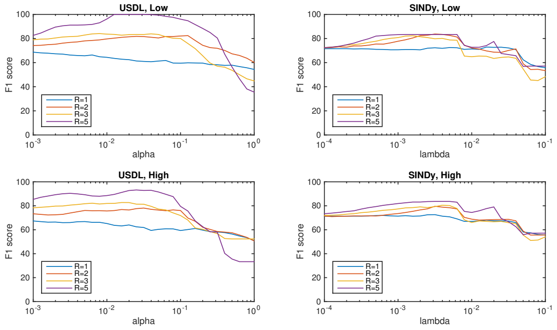

(a) F1 scores

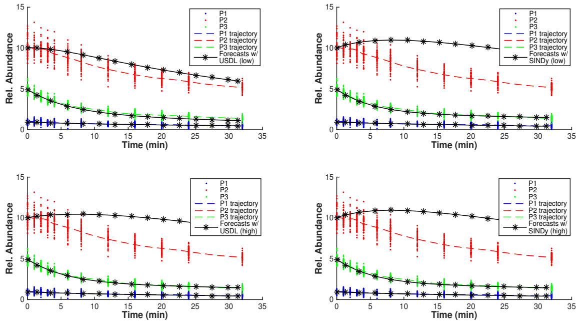

(b) Time-series forecasts

Figure 1: (a) The F1 score as a function of the hyperparameter for both USDL (left column) and SINDy (right column) and various number of experiments and noise levels. (b) Time-series forecasts (black curves) based on the estimated parameter values for both USDL (left column) and SINDy (right column).

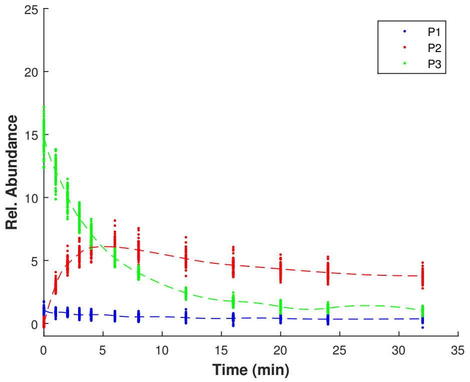

(a) Time-course measurements and time-series interpolation

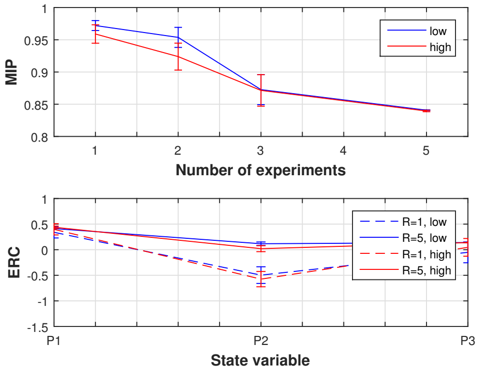

(b) MIP and ERC

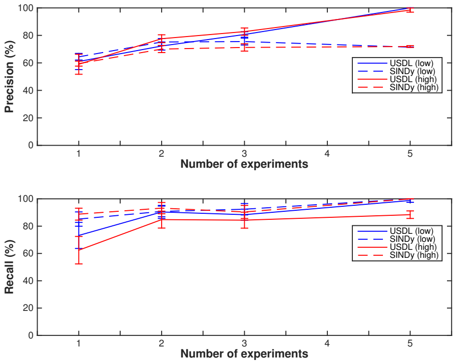

(c) Precision and recall

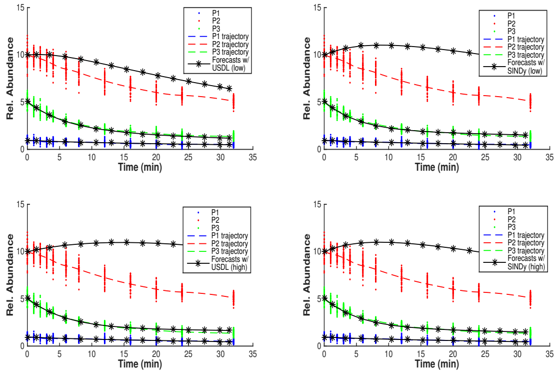

(d) Time-series forecasts

Figure 2: (a) Time-series interpolation for the protein interaction network when the smoothing weight parameter is $\lambda_C = 1$. Trajectories are not smooth introducing further noise, nevertheless, the dynamics of the measurements are correctly captured. (b) MIP (upper panel) and ERC per state variable (lower panel) for two noise levels; low (blue) and high (red). ERC is negative when $R = 1$ experimental condition (dashed lines) is considered while it is positive when $R = 5$ experimental conditions (solid lines) are considered. (c) Precision (upper panel) and recall (lower panel) curves as a function of R for both USDL (solid lines) and SINDy (dashed lines) algorithms. Perfect reconstruction is achieved for the low noise level and when $R = 5$ interventions are provided. (d) Time-series forecasts (black curves) based on the estimated parameter values for both USDL (left column) and SINDy (right column). Forecasts are similar to the case where $\lambda_C = 100$.

1.1 Further exploration

We proceed by showing the results on the performance of the proposed approach under various experimental conditions. We test the performance when the number of sampling points is reduced as well as when different weights for the smoothing penalty in the collocation method are shown next. In all experiments of this subsection, we set the hyperparameter of both USDL and SINDy algorithms based on the optimal F1 score value. We do not show these plots since they are very similar to Figure 1(a). Figure 2 presents the interpolated time-series of each species when $\lambda_C = 1$. It is evident with naked eye that the bending of the trajectories are harsher, especially, near the sampling points. Nevertheless, positive values of ERC for all species when all five interventions are taken into account indicate the perfect reconstruction of the protein interaction network. Indeed, the performance of the USDL algorithm measured by precision/recall metrics and forecast capacity on new experiment is only slightly affected as shown in Figures 2(c) & (d), respectively. SINDy algorithm, as in the main text, does not exploit the information from the multiple interventions. Thus, SINDy's performance is suboptimal. On the opposite direction where smoothing weight is large ($\lambda_C = 10^4$), the constructed trajectories are fairly smoother as Figure 3 shows. Despite the error on the dynamics, the performance of the USDL algorithm is not negatively affected as precision and recall plots reveal. Evidently, the accuracy of both algorithms is slightly improved for the high level noise with USDL achieving perfect reconstruction when fed with data from five interventions. It is noteworthy that ERC indicates this behavior since it is larger under high noise (red solid line in Figure 3(b)). In both cases the inference methods where robust against the hyperparameter of the collocation method.

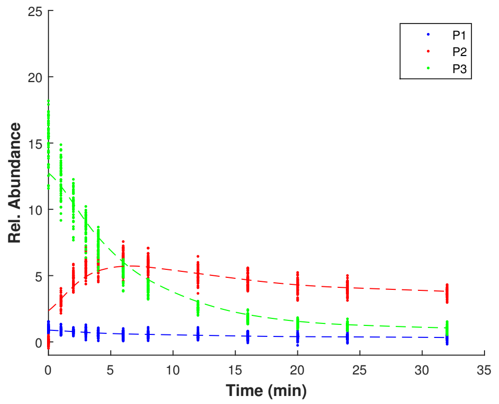

(a) Time-course measurements and time-series interpolation

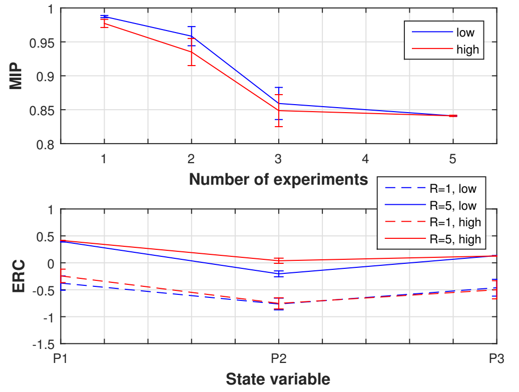

(b) MIP and ERC

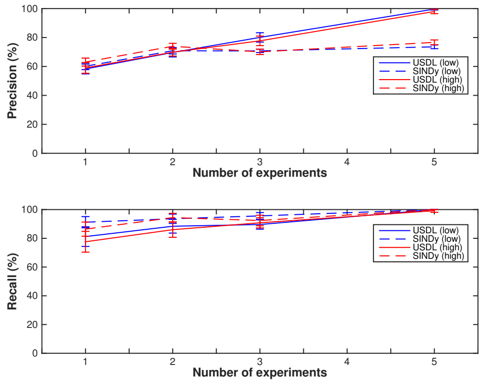

(c) Precision and recall

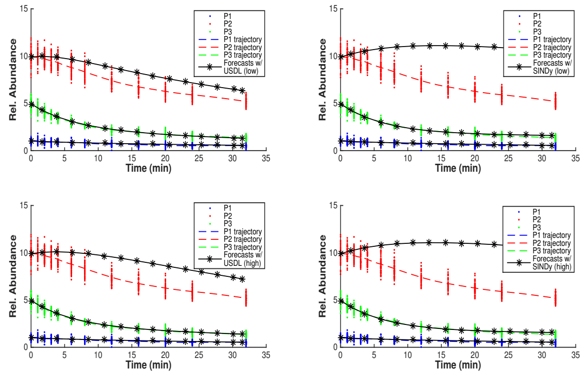

(d) Time-series forecasts

Figure 3: (a) Time-series interpolation for the protein interaction network when the smoothing weight parameter is $\lambda_C = 10^4$. Trajectories are excessively smoothed resulting in incapability of capturing correctly the dynamics. (b) MIP (upper panel) and ERC per state variable (lower panel) for two noise levels; low (blue) and high (red). (c) Precision (upper panel) and recall (lower panel) curves as a function of R for both USDL (solid lines) and SINDy (dashed lines) algorithms. Perfect reconstruction is achieved for USDL algorithm at both low and high noise levels. (d) Time-series forecasts (black curves) based on the estimated parameter values for both USDL (left column) and SINDy (right column).

The next experiment assesses the performance of the proposed algorithm when measurements from fewer time-points are obtained. Since dense sampling is more expensive both in time and in money than sparse sampling, it is desirable the learning methods be robust against less sampling points. Figure 4 presents the results when we reduce the number of

sampling times by a factor of 3 (from 12 to 4) and keeping the time instants at 0,2, 8 and 32. Interestingly, the performance of the USDL algorithm remained the same with perfect reconstruction being achieved when all five interventions are considered under low noise. This implies that the collocation method was able to correctly estimate the trajectories from these four time points. Perfect reconstruction was also predicted from the positivity of the ERC for all variables for the case of five interventions. We would like to remark here that ERC can be utilized as an experimental design metric for the determination of the optimal sampling points. Indeed, using prior information about the dynamics of the system under study, the sampling points space could be explored. Optimal conditions may be achieved by making ERC as large as possible for all variables. Additionally, the number as well as the type of interventions would be chosen based on the ERC, however, this is a different study which we leave it as future work.

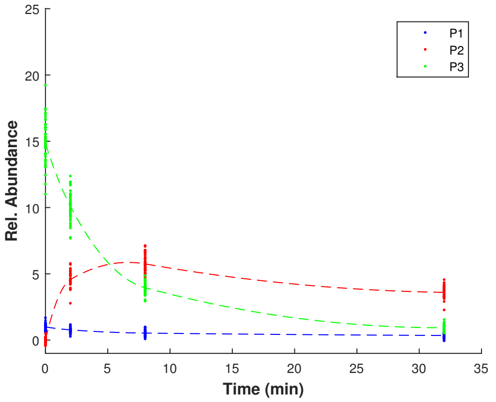

(a) Time-course measurements and time-series interpolation

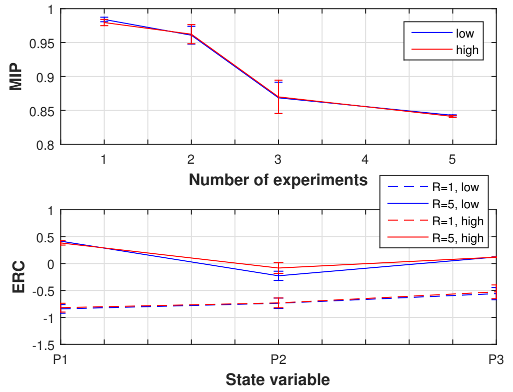

(b) MIP and ERC

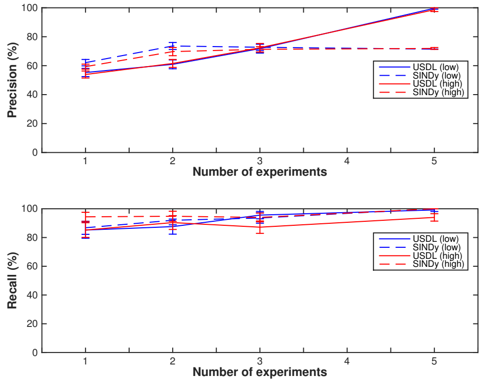

(c) Precision and recall

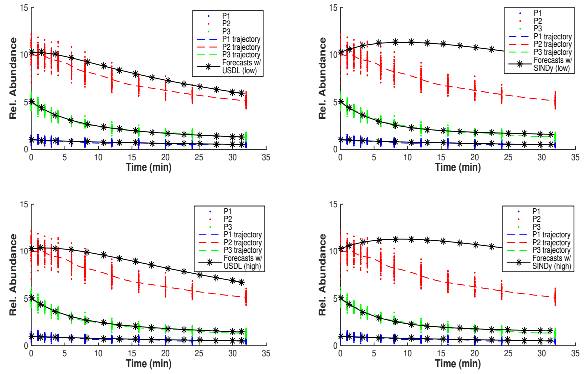

(d) Time-series forecasts

Figure 4: (a) Time-series interpolation for the protein interaction network when less sampling points are measured. (b) MIP (upper panel) and ERC per state variable (lower panel) for two noise levels; low (blue) and high (red). ERC is positive when $R = 5$ interventions are considered (solid lines) while it is negative when $R = 1$ intervention is considered (dashed lines). (c) Precision (upper panel) and recall (lower panel) curves as a function of R for both USDL (solid lines) and SINDy (dashed lines) algorithms. Overall, the right positioning of the sampling instants resulted in robust inference of the network of interactions. (d) Time-series forecasts (black curves) based on the estimated parameter values for both USDL (left column) and SINDy (right column).

The final experiment assesses the performance of the proposed algorithm when the protein interaction network has different reaction constants. Nature rarely has the favourite constant values for an algorithmic approach and it is important to test it under different conditions. Figure 5 presents the results when the reaction constants of the first two reactions are doubled (i.e., $k_1 = 8$ and $k_2 = 0.1$). Even though the reduction of MIP, the perfect reconstruction of the network is lost and possibly more than five interventions are required in order to achieve true recovery of the protein interaction network. Interestingly, ERC is not positive for all variables with the problematic species being P_2 whose ERC is negative. For the case where all five interventions are taken into account, the inspection of the estimated connectivity matrix (i.e., of A) revealed that the row that describes the dynamics of P_2 was most of the times wrong (more than 95% wrong) while the other two rows that describe the dynamics of P_1 and P_3 were most of the times correct (more than 95% correct). Nevertheless, the total error in the structure inference significantly affect the forecast accuracy of all variables

as Figure 5(d) demonstrates.

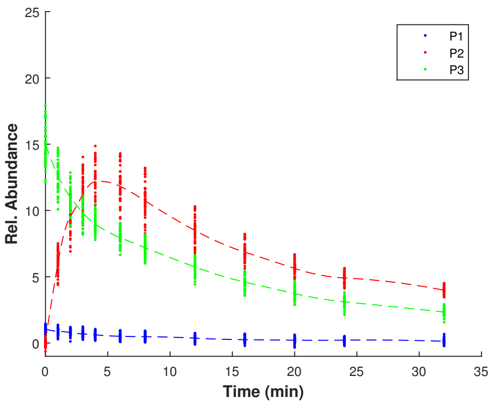

(a) Time-course measurements and time-series interpolation

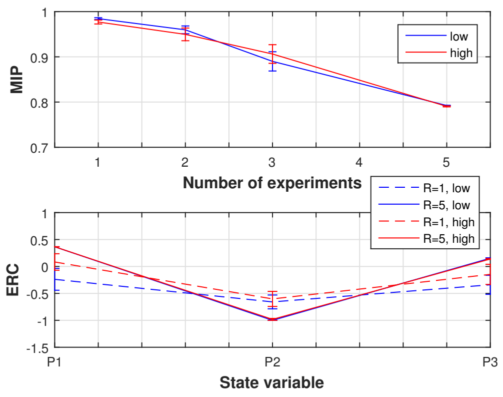

(b) MIP and ERC

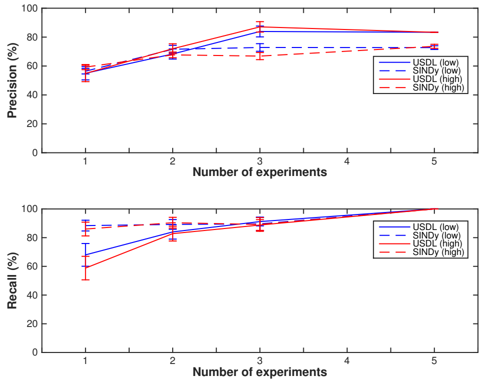

(c) Precision and recall

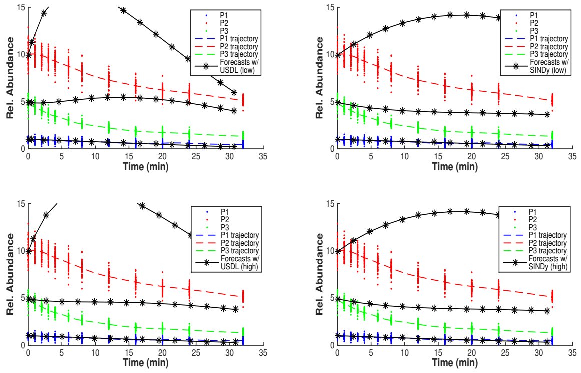

(d) Time-series forecasts

Figure 5: (a) Time-series interpolation for the protein interaction network when different reaction constants are assumed without changing the topology of the network. (b) MIP (upper panel) and ERC per state variable (lower panel) for two noise levels; low (blue) and high (red). ERC is negative for P_2 even when $R = 5$ interventions are considered (solid lines). (c) Precision (upper panel) and recall (lower panel) curves as a function of R for both USDL (solid lines) and SINDy (dashed lines) algorithms. Performance results indicate that perfect reconstruction is not achieved at any case which was again expected from the negativity of ERC for P_2. Perfect network reconstruction requires more interventions. (d) Time-series forecasts (black curves) based on the estimated parameter values for both USDL (left column) and SINDy (right column). The forecast error for P_2 is larger for this case confirming once again that ERC nicely correlates with the forecast outcome.

Overall, this example demonstrates that when ERC is positive for all variables then with high probability the protein interaction network will be perfectly reconstructed. In contrast, when there are variables whose ERC is negative then the probability of perfect reconstruction becomes very low and more experiments are required in order to make ERC positive and thus recover the true network of interactions. Since ERC is a variable-dependent quantity, we can be confident that the found interactions between variables with positive ERC are true. Finally, we remark that for small systems of interactions like this one, a brute force alternative is feasible. A complete search of all possible solutions when the non-zero components are less than ten is computationally tractable for dictionary size up to twenty atoms. However, such an approach will provide little or no information on how to design a new experiment or a new data acquisition policy compared with greedy algorithms or convex relaxation methods where metrics such as MIP and ERC can guide the experimental designer.

2 Mass Cytometry

The protein interactions found in the literature are reported on Table 2. In the right most column, the scientific source of the corresponding interaction is provided. The smoothing weight in collocation method is $\lambda_C = 10^4$. In order to make the results robust, we repeat the inference 100 times by using half of the points in each iteration. An edge is added to the

network when it is found at least 80% of the times. The hyperparameter tuning is based on the optimal F1 scores shown in Figure 6.

Table 2: Protein interactions found in the literature.

Interaction	Literature
CD3z → Slp76	Krishnaswamy et al. [2]
Slp76 → Erk	Krishnaswamy et al. [2]
Erk → S6	Krishnaswamy et al. [2]
Erk → Creb	Krishnaswamy et al. [2]
CD3z → MAPKAPKII	Krishnaswamy et al. [2]
MAPKAPKII → Creb	Krishnaswamy et al. [2]
Akt → Rb	Krishnaswamy et al. [2]
Akt → S6	Krishnaswamy et al. [2]
Akt → Creb	PI3K-AKT Signaling (KEGG)

Table 3 reports the value of MIP and ERC per variable for the two subnetworks presented in the main text. MIP is almost 1 showing that there is strong collinearity between the columns of dictionary matrix, Ψ. ERC values for the small subnetwork are positive increasing the confidence that the inferred network is correct. On the contrary, some variables have negative ERC for the larger subnetwork casting doubt on the correctness of the inferred network.

Table 3: MIP and ERC values for the two subnetworks of proteins. Positive ERC adds confidence to the inference results, however, it is just an estimate since the ground truth is unknown.

	MIP	ERC							
	–	CD3z	SLP76	Erk	S6	MAPKAPKII	Creb	Akt	Rb
Subnet 1	0.967	0.032	0.114	0.020	0.065	–	–	–	–
Subnet 2	0.980	0.031	0.119	-0.314	-0.256	-0.163	0.031	0.119	-2.545

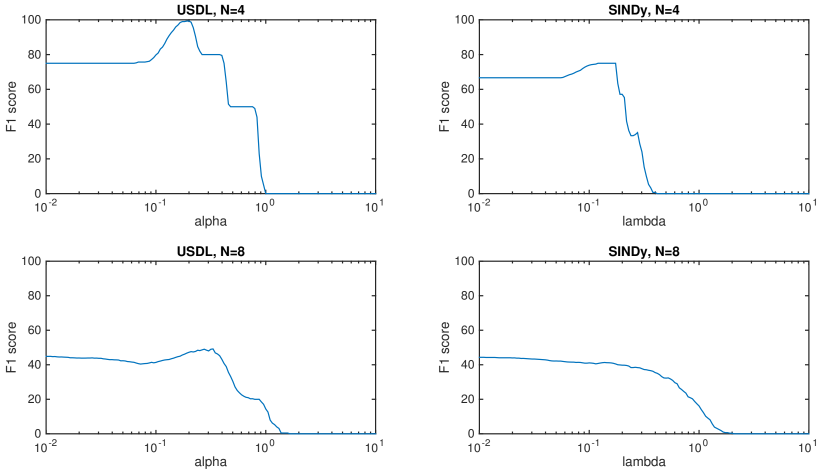

Figure 6: The F1 score as a function of the hyperparameter for both USDL (left column) and SINDy (right column). The first row shows the F1 score for the four protein subnetwork while the second row for the eight protein subnetwork. Evidently, F1 score decreases as the number of proteins is increased for both approaches.

Next, we examine the inference capabilities of the proposed approach under various experimental conditions. Figure 7 presents the first experiment where we eliminate half of the sampling points. The new sampling points are at 0, 2, 5, 8, 20 minutes. As Figure 7(a) asserts the network inference with USDL deteriorates since the interaction CD3z → Slp76 is missed while an additional interaction that Slp76 produces S6 is found. SINDy is minimally affected by the elimination of the half measurements inferring though an almost fully-connected graph. For the eight-protein network, the inference deteriorates when less sampling points are present for both USDL and SINDy (see Figure 7(c) & (d), respectively).

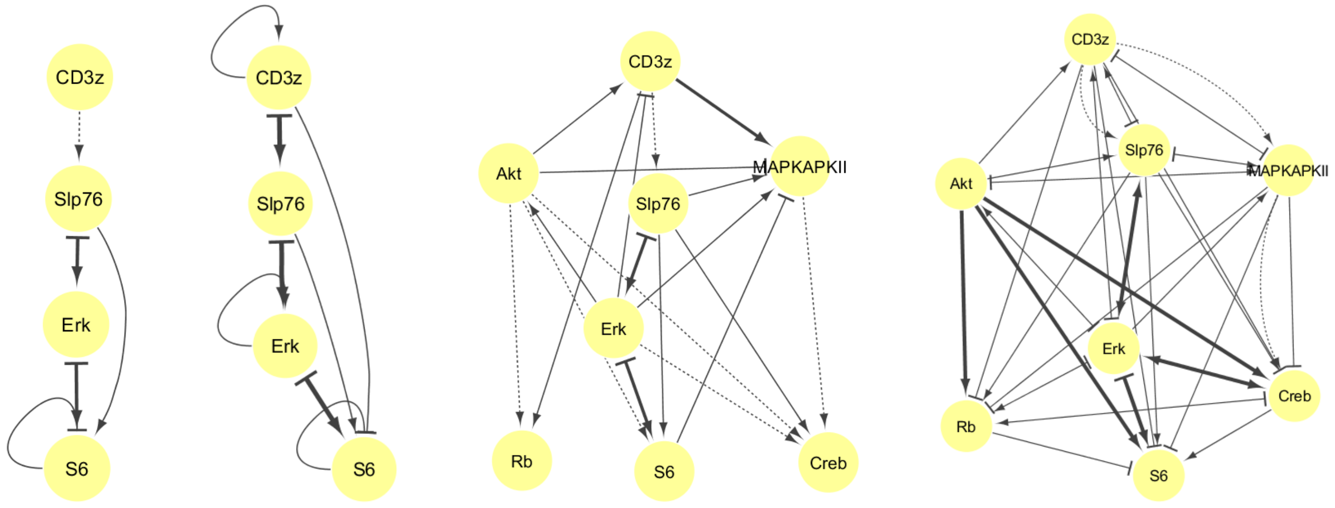

(a) USDL (b) SINDy (c) USDL (d) SINDy

Figure 7: Reconstructed protein signalling networks when half of the sampling points are provided.

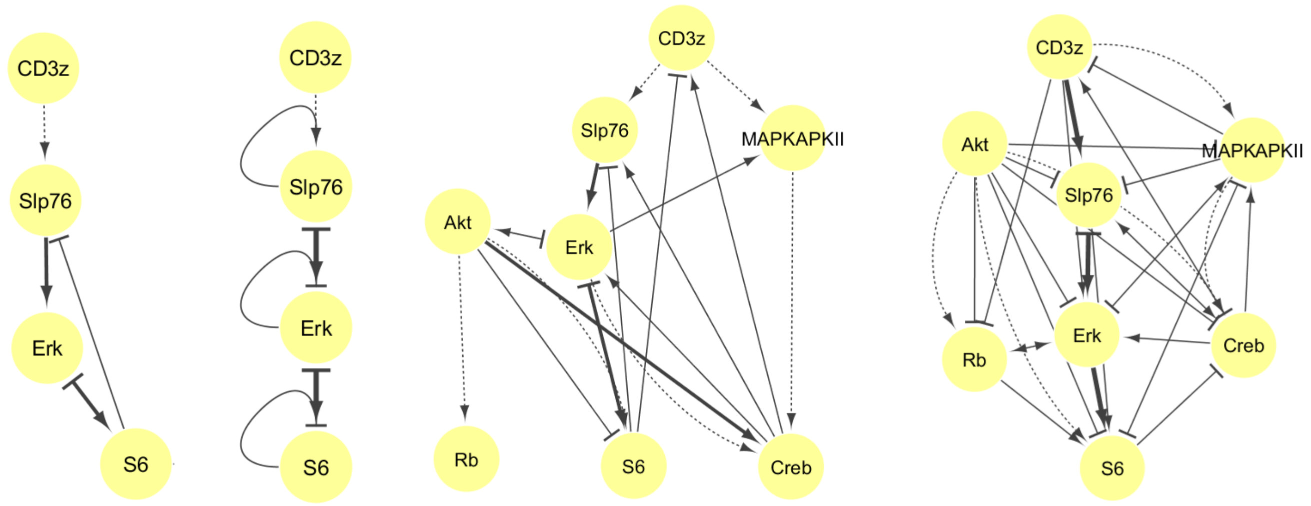

(a) USDL (b) SINDy (c) USDL (d) SINDy

Figure 8: Reconstructed protein signalling network when both activation cocktails are provided.

Figure 8 demonstrates the reconstructed networks when both activation cocktails are taken into consideration. Again, network inference deteriorates for both algorithms even though we provide additional data from another intervention. A probable explanation is that there exist interference between measured and unmeasured proteins with the unmeasured ones serve as latent variables. These extra sources of noise confuse the inference algorithm resulting in inferior performance. We also tested the performance when all three available populations are taken into consideration and fed to the inference algorithms and the results are presented in Figure 9. One activation cocktail (CD3/CD28) is considered. For the USDL algorithm there are few edges that are missing. On the contrary, SINDy algorithm especially for the eight-protein network is able to reconstruct most of the interactions with a minimum number of false positives. This is a rather unexpected result since different populations have different variable connectivity.

The final experiment is to add all the available measurements with all three populations and both activation cocktails (Figure 9). The results as quantified by the reconstructed networks are similar to the case where both activation cocktails and one population (i.e., CD4 naive) is considered. Overall, we conclude that more data does not always produce superior results and caution is necessary in the experimental design in order to reveal the actual protein interactions. Interestingly, the worst performance results for both inference algorithms are obtained when all activators are considered . In this case, only two protein interactions that have been reported in the literature are found. It is also noteworthy that there are two interactions that are present in all settings of the experiments. These interactions form the cascade Slp76 $\rightarrow$ Erk $\rightarrow$ S6. Moreover, in all experiments with USDL, the edge between Erk and Akt is always inferred which can be either a true interaction not reported or a way to up-regulate Akt since in the T-cell signaling pathway Akt is phosphorylated by CD28

which is not measured.

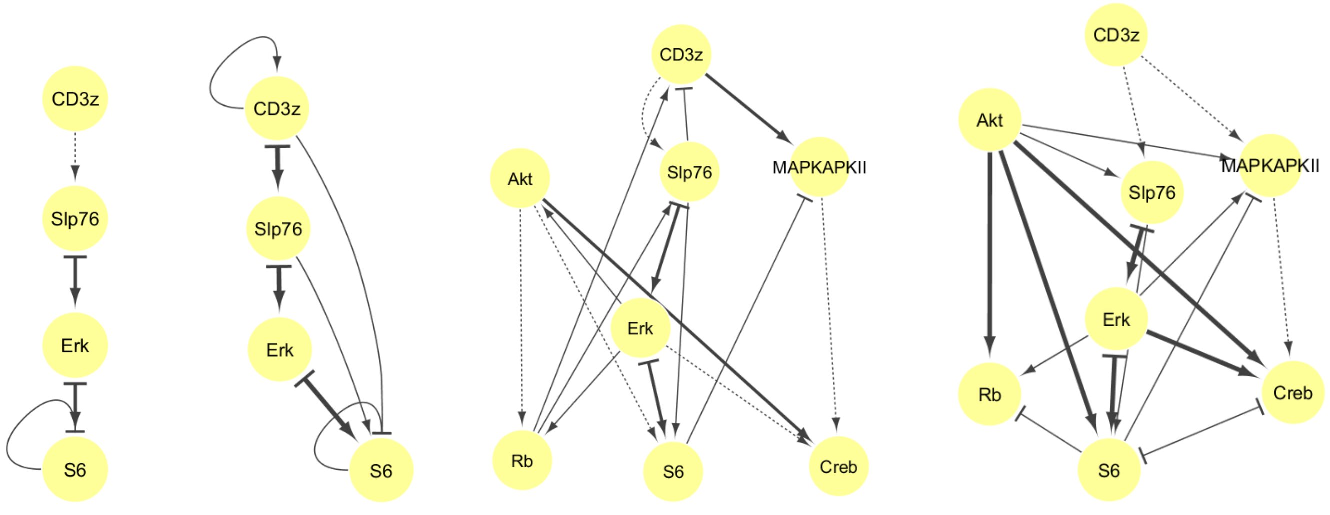

(a) USDL (b) SINDy (c) USDL (d) SINDy

Figure 9: Reconstructed protein signalling network when three dinstict populations are provided.

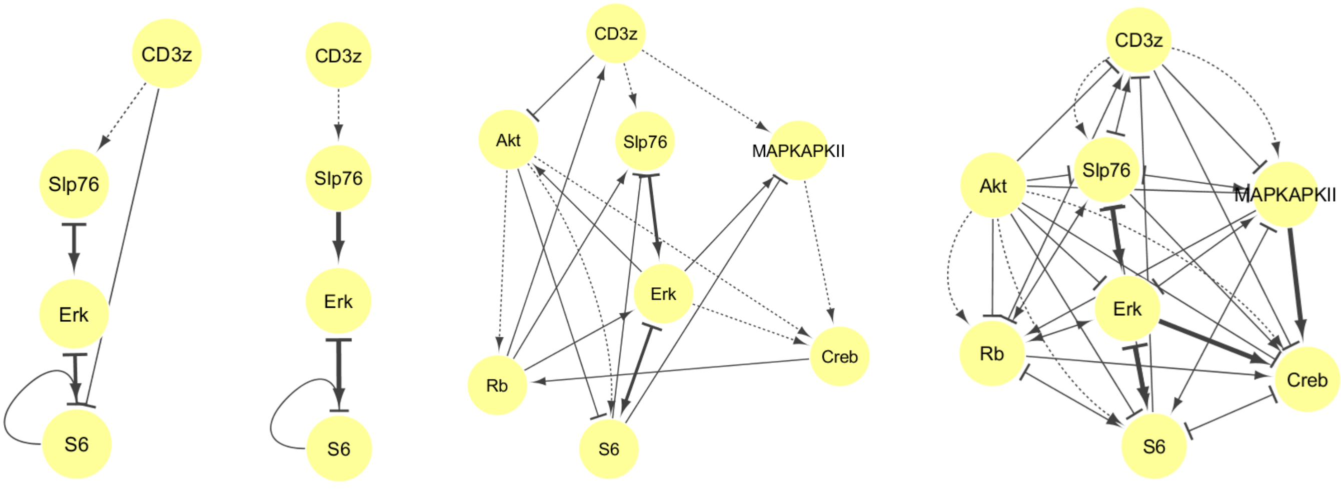

(a) USDL (b) SINDy (c) USDL (d) SINDy

Figure 10: Reconstructed protein signalling network when all available data are provided.

Finally, we present an experiment where prior knowledge is provided to the inference algorithm. It is legitimate to use any available prior information to guide the inference algorithm especially when a scientist is searching for new knowledge. Our algorithm and in particular OMP can easily incorporate prior knowledge as discussed earlier. Thus, one can provide the known interactions and look for new ones. In Figure 11 we present the reconstructed network when the following interactions are a priori provided: CD3z $\rightarrow$ Slp76, CD3z $\rightarrow$ MAPKAPKII, MAPKAPKI I $\rightarrow$ Creb and Erk $\rightarrow$ Creb. There are two immediate observations related with Creb that can be made. First, the interaction Akt $\rightarrow$ Creb is lost probably because it has been replaced by Erk $\rightarrow$ Creb and, second, instead of up-regulating Creb, MAPKAPKII down-regulates it questioning the existence of this interaction.

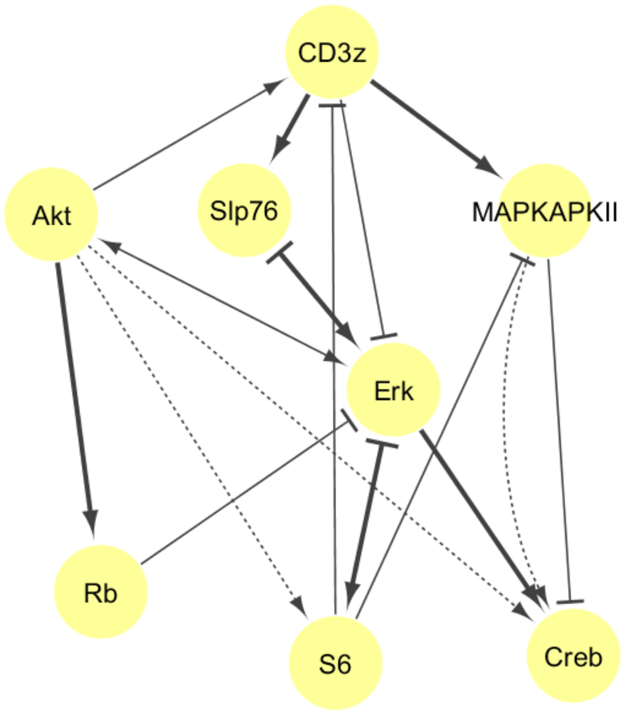

Figure 11: Reconstructed eight-protein subnetwork when prior knowledge is added. Measurements are from CD4+ naive cells with CD3/CD28 activation cocktail.

3 Multidimensional Ornstein-Uhlenbeck process

The multidimensional Ornstein-Uhlenbeck (OU) process is defined in the more standard notation as

$$dx_t = -Ax_t dt + \sigma dB_t \tag{1}$$

where A is the connectivity matrix, B_t is an N-dimensional standard Brownian motion while σ is the noise level which is set to 0.25. The OU process satisfies the detailed balance condition [3] thus, at the stationary regime, it is time-reversible meaning that the reflected time-series have the same path distribution. Moreover, the stationary distribution, $\mu(x)$, is proportional to

$$\mu(x) \propto e^{-\frac{1}{\sigma^2}x^T\Sigma^{-1}x} . \tag{2}$$

Obviously, it is a zero-mean Gaussian. The covariance matrix Σ is found as the solution of the Lyapunov equation $A\Sigma + \Sigma A^T + I = 0$. Concentration matrix which is defined as the inverse of the covariance matrix is not necessarily sparse even when A is sparse. This implies that if the measurements are obtained as i.i.d. samples from the stationary distribution then it is impossible to infer the causal relationships between the state variables since they have been lost. On the contrary, using the richer information that is contained by the dynamics, primarily time correlations, the true connectivity matrix is estimated and the causal relations can be correctly inferred.

Proceeding, the time-series of an $N = 20$-dimensional Ornstein-Uhlenbeck (OU) process are presented in Figure 12(a). The upper panel shows the time-series at the stationary regime while the lower panel shows the time-series at the transient regime. At the transient regime, the initial values of the process were independently sampled from a Gaussian distribution with variance one which obviously result to starting the process out of equilibrium. The OU process converges to equilibrium after one time unit. The numerical integration is performed using Euler-Maruyama scheme which is a first-order finite difference scheme with time step of $\Delta t = 0.001$. The results of Fig. 3 in the main text are produced by setting the maximum allowed non-zero elements in USDL algorithm to be $K = 6$ while the threshold is set according to the maximum value of the F1 score which is shown in Figure 13(a). SINDy algorithm's hyperparameter is similarly set. Figure 13(a) also reveals that there is a large region of hyperparameter values that produce perfect reconstruction at the transient regime. The region of optimal values is much smaller at the stationary regime but as we increase the number of time-series it tends to increase at least for the USDL algorithm. Additionally, this figure highlights the significant superiority of the USDL algorithm over SINDy algorithm when the noise is prominent (i.e., at the stationary regime) showing the generality of the proposed methodology.

Figure 12(b) shows ERC values per variable. It is evident that when $P = 5$ time-series are fed to USDL algorithm (dashed lines), ERC is negative in both regimes for all variables. Moreover, ERC for the transient regime is worse that

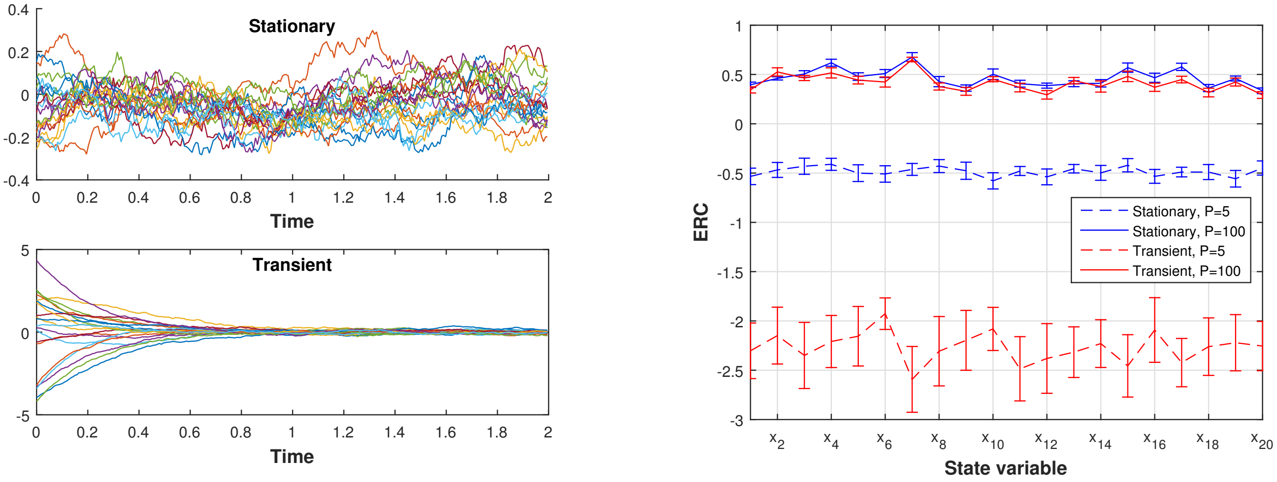

(a) Time-series for both regimes (b) ERC per state variable

Figure 12: (a) Time-series of a multi-dimensional OU process at stationary regime (upper plot) as well at transient regime (lower plot). (b) ERC for each variable of the OU process at both regimes and two different number of trajectories. ERC is positive when $P = 200$ time-series are given (solid lines).

the stationary regime which might be caused due to different threshold values. When $P = 100$ time-series are fed to the inference algorithm then ERC at both regimes is positive for all variables and ERC take slightly larger values in the transient regime. Nevertheless, as it is evident from the precision-recall curves (Fig. 2(b) in the main text where precision is slightly above 50%), perfect reconstruction is not achieved with stationary time-series. This is a consequence of the fact that the strength of the interaction coefficients (i.e., non-zero elements of A) compared to the noise level is low. Indeed, noise is the primal driving force of the dynamics in the stationary regime thus it is harder to infer accurately the connectivity matrix. Only when $P = 1000$ the signal-to-noise ratio is high enough for perfect reconstruction of the dynamical system.

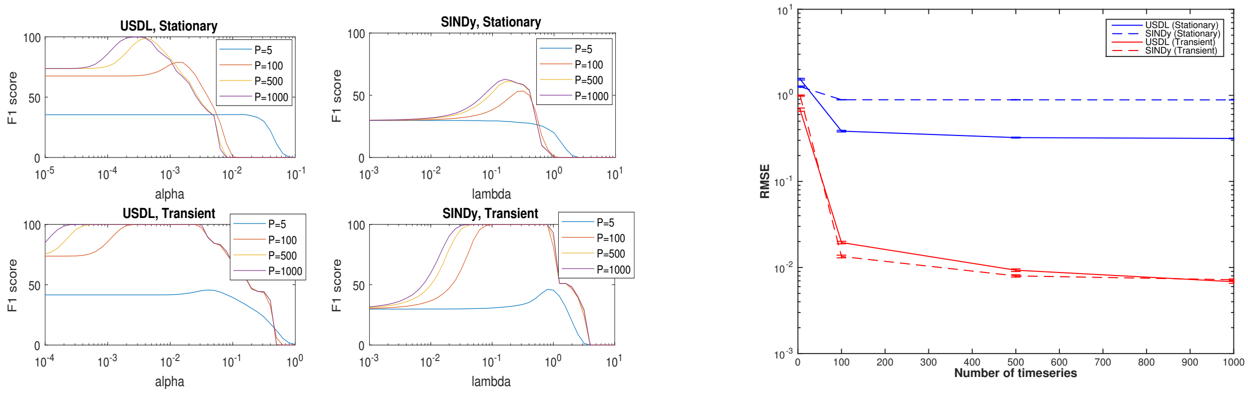

(a) F1 score (b) RMSE

Figure 13: (a) The F1 score as a function of the hyperparameter for both USDL (left column) and SINDy (right column) and various number of time-series using the peaky Fourier modes as test functions. (b) The respective RMSE for both USDL (solid lines) and SINDy (dashed lines). The parameter estimation is more accurate at the transient regime (almost two orders of magnitude) due to the higher signal-to-noise ratio compared to the stationary regime.

Figure 13(b) presents the RMSE results for the parameter estimation of the connectivity matrix A. RMSE results are qualitatively similar to the precision-recall results since RMSE at the transient regime is two orders of magnitude less that the RMSE at the stationary regime. Furthermore, USDL performs better at the stationary regime than SINDy revealing that a well-educated selection of the family of test functions could also improve the parameter estimation outcomes.

3.1 Selection of Test Functions

In the main text, we highlight that the crucial decision is related with the definition of the test functions. Figure 14 presents the results of USDL algorithm when $M = 81$ Fourier modes are deployed. Both precision and recall are about 90%. As in all other experiments, we set USDL algorithm's threshold according to the optimal F1 score shown in Figure 15(a). Results from SINDy algorithm are shown for comparison purposes. Looking in depth why perfect reconstruction was not successful, we found out that the problematic cases had the following pattern. When an interaction of the form $x_n \to x_{n'}$ exist in the connectivity matrix then OMP inferred two interactions; the correct one as well as that $x_{n'} \dashv x_n$. These two types of interaction are closely related since they both assert that x_n precedes $x_{n'}$. The problematic inference arises because the degradation of x_n, which is represented as $x_n \dashv x_n$ is not enough to explain the dynamics of x_n hence additional interactions are inferred. These additional edges are not random but they represent variables with strong time correlations. A way to separate these additional interactions is to observe that the time cross-correlations between one variable and the other variables are ordered based on which are the driving forces. Thus, we need to define another type of test functions with the property of sharp changes which will be able to separate between small time-differences. For instance, multiplying the Fourier modes with sawtooth functions would work. However, sawtooth function is not smooth thus we propose to use the following test functions which we call peaky Fourier modes

$$\phi_{2m-1}(t) = \frac{cos(4\pi mt/T)}{0.01 + cos(4\pi mt/T)^2} \cos(2\pi mt/T) \tag{3}$$

and

$$\phi_{2m}(t) = \frac{cos(4\pi mt/T)}{0.01 + cos(4\pi mt/T)^2} \sin(2\pi mt/T) \tag{4}$$

with $m = 1, ..., (M-1)/2$. We remark that the above functions are infinitely smooth but they have abrupt edges. Figure 16 show the sine and cosine Fourier modes for $m = 5$ (blue lines) as well as the respective sine and cosine peaky Fourier modes (red lines).

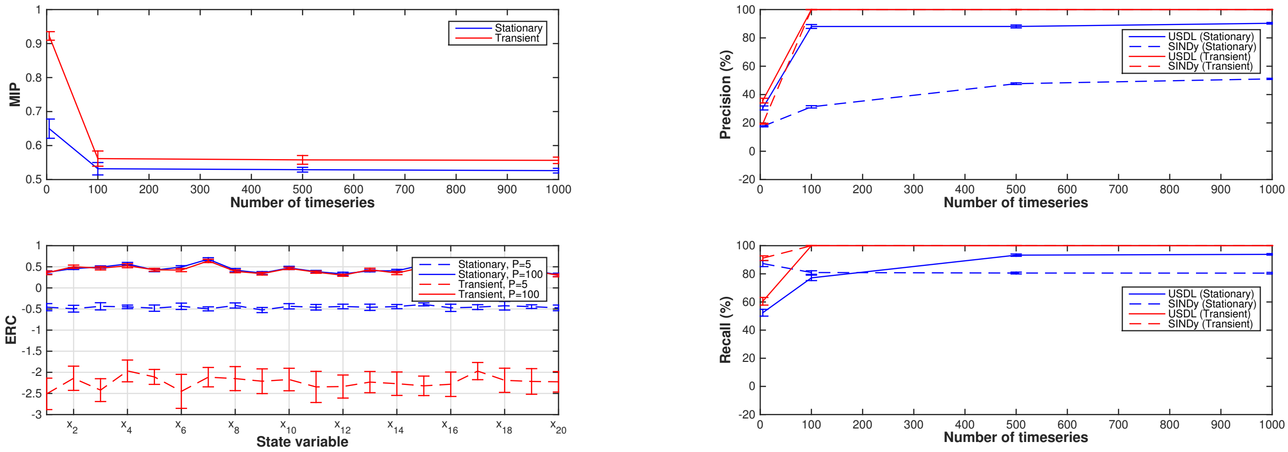

(a) MIP and ERC

(b) Precision and recall

Figure 14: (a) MIP (upper panel) and ERC per state variable (lower panel) for the OU process when more test functions are considered. The number of Fourier modes is $M = 81$. (b) Precision (upper panel) and recall (lower panel) curves as a function of P, i.e., the number of trajectories for both USDL and SINDy algorithms. However, perfect reconstruction is not achieved.

References

[1] R. N. Gutenkunst, J. J. Waterfall, F. P. Casey, K. S. Brown, C. R. Myers, and J. P. Sethna. Universally sloppy parameter sensitivities in systems biology models. *PLOS Computational Biology*, 3, 2007.

[2] Smita Krishnaswamy, Matthew H. Spitzer, Michael Mingueneau, Sean C. Bendall, Oren Litvin, Erica Stone, Dana Pe'er, and Garry P. Nolan. Conditional density-based analysis of T cell signaling in single-cell data. *Science*, 346(6213), 2014.

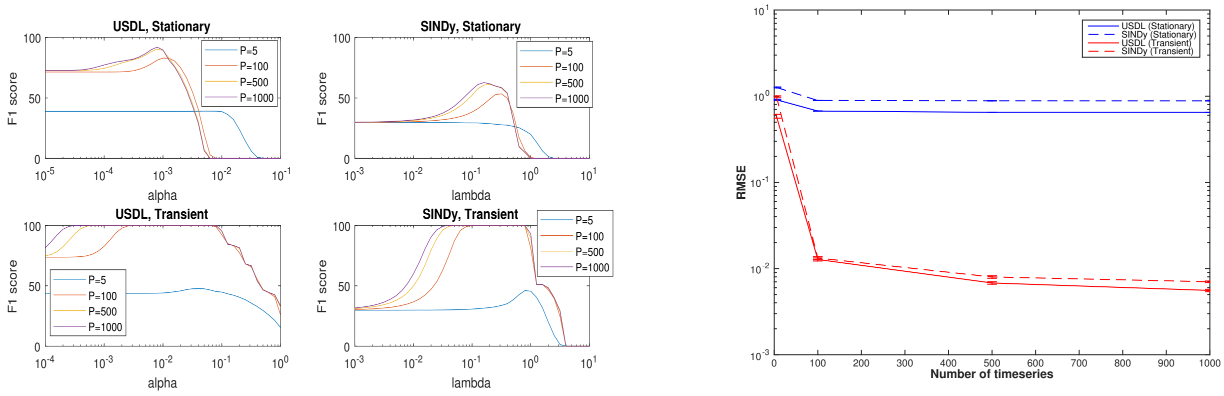

(a) F1 score (b) RMSE

Figure 15: (a) The F1 score as a function of the hyperparameter for both USDL (left column) and SINDy (right column) and various number of time-series using the standard Fourier modes. (b) The respective RMSE for both USDL (solid lines) and SINDy (dashed lines).

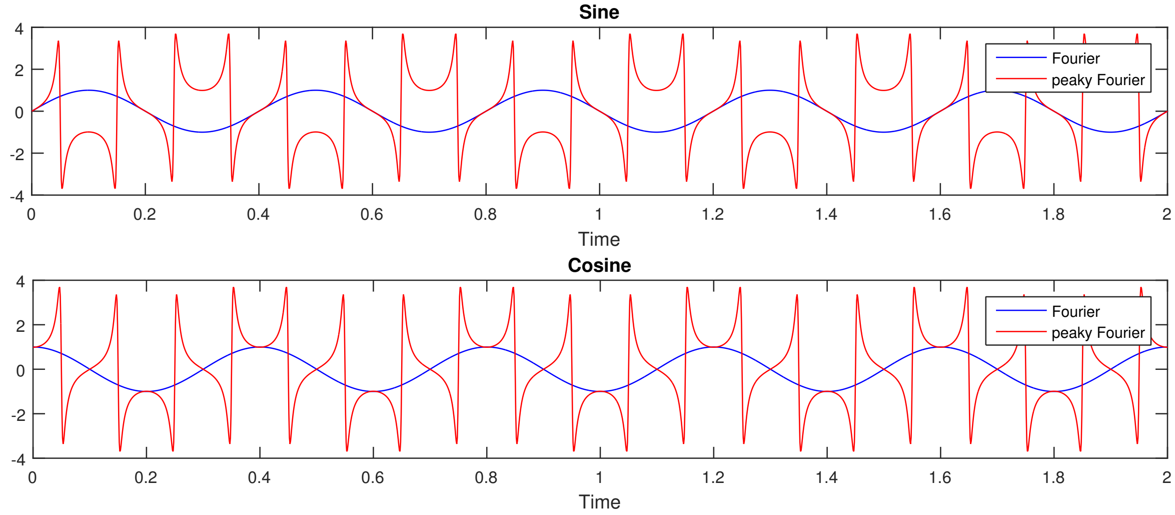

Figure 16: Upper plot: Sine Fourier mode (blue) and its peaky version (red). Lower plot: Same as upper plot but for cosine Fourier mode. Notice that peaky Fourier modes are actually smooth but they do have abrupt changes. The advantage of peaky Fourier as test functions is that they "extract" information in an adequate manner as they capture variations more accurately.

[3] Crispin Gardiner. *Handbook of stochastic methods: for physics, chemistry & the natural sciences*. Springer-Verlag, 2004.
