# Supplementary material for: A unified approach for sparse dynamical system inference from temporal measurements
Source: Bioinformatics. 2018 Jan 31;35(18):3387–96. doi: 10.1093/bioinformatics/btz065 (PMC6748758; doi:10.1093/bioinformatics/btz065)
Supplement: btz065_Supplementary_Materials [file btz065_supplementary_materials.zip › btz065-suppl_data/SI6_Text.pdf]

Sparse Learning of the Lorenz96 Model

Yannis Pantazis and Ioannis Tsamardinos

Introduction

Lorenz96 [1] is an idealized deterministic climate model whose nonlinear dynamical set of equations is given in Figure 1(a). We set $N = 10$ and for large enough force ($F \geq 8$) the system is chaotic (Figure 1(b)). The time-series of Lorenz96 system are generated through numerical integration. We utilized a third-order Runge-Kutta scheme with time step set to 0.001. The initial values for each time-series are randomly and uniformly sampled in the interval $[-F/2, F/2]$. The sampling rate is set to $1000Hz$ or, equivalently, the sampling time is 0.001 which is low enough so as to assume that the complete time-series is measured.

$$
\begin{aligned}
\dot{x}_1 &= F - x_1 + x_N x_2 - x_N x_{N-1} \\
\dot{x}_2 &= F - x_2 + x_1 x_3 - x_1 x_N \\
\dot{x}_3 &= F - x_3 + x_2 x_4 - x_2 x_1 \\
&\dots \\
\dot{x}_N &= F - x_N + x_{N-1} x_1 - x_{N-1} x_{N-2}
\end{aligned}
$$

(a) Lorenz96 system

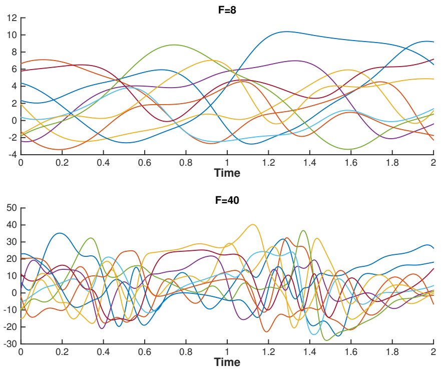

(b) Lorenz96 trajectories

Figure 1: (a) The nonlinear system of equations with periodic indexing. F is the driving force, the linear term corresponds to the dissipation of energy while the quadratic terms correspond to the convection (mixing). Force values above 8 results in chaotic behavior [1]. (b) Time-series (or trajectories) of Lorenz96 for two values of F. Upper plot shows the time-series under weak force ($F = 8$) while the lower plot shows the time-series under strong force ($F = 40$).

In order to perform sparse learning inference, the dictionary elements have to be determined. The chosen dictionary, $\psi(x)$, contains the constant term, the linear terms and all quadratic combinations resulting in $Q = 66$ dictionary atoms (or constructed features) in total. Then, the elements of vectors z_n with $n = 1, ..., N$ and matrix Ψ are numerically estimated using the trapezoidal rule. Even though this is a deterministic system and should fall to the noiseless SSR case, this is not true since there are errors, first, in the construction/discretization of the time-series due to the numerical scheme and, second, in the numerical evaluation of the integrals. We tested two cases one with the force taking a low value ($F = 8$) and another case taking a high value ($F = 40$). In total, $M = 41$ Fourier modes which constitutes of the constant function, 20 sines and 20 cosines in the interval $[0, 2]$ were defined as test functions.

Figure 2 summarizes the performance of USDL approach as a function of the number of time-series denoted by P. Threshold value for OMP algorithm was set to $\alpha = 0.1$ while the maximum number of non-zero components was set to $K = 7$ which is larger than the true value which is 4. As quantified from the precision-recall subplots (see Figure 2(a)), the performance in terms of both precision and recall is improved as the number of measured time-series is increased. It actually reaches perfect reconstruction when $P = 5$ for the strong forcing case and when $P = 20$ for the weak forcing case. In general, stronger forces which result in more chaotic behavior and stronger mixing are helpful in identifying the true model as it is evident from the fact that red curves outperformed the blue ones. Figure 2(a) presents also the reconstruction accuracy of SINDy algorithm [2] (dashed lines) with its hyperparameter value being set to $\lambda = 0.1$. We employ the central difference scheme which is a second order

method for the numerical estimation of the derivatives. Interestingly, SINDy requires less time-series in order to achieve perfect reconstruction in both parameter regimes showing that Lasso is a competitive alternative for solving the SSR problem.

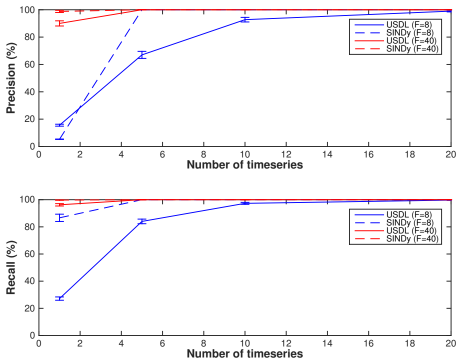

(a) Precision and recall

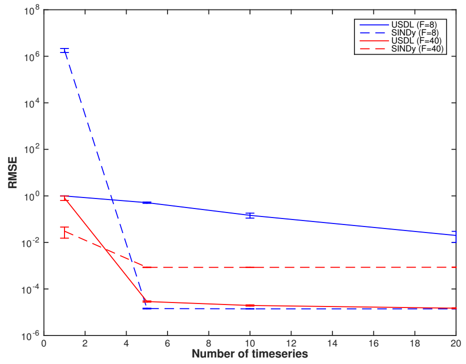

(b) RMSE

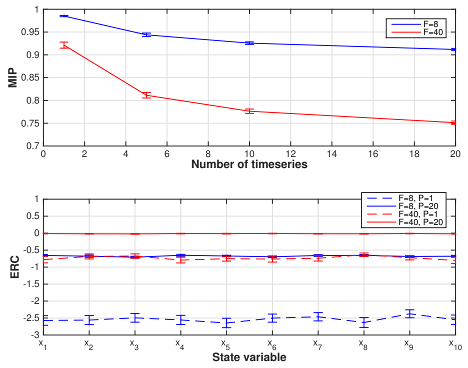

(c) MIP and ERC

Figure 2: Performance analysis of USDL algorithm for the nonlinear Lorenz96 climate model. (a) Precision and recall curves under two different parameter regimes; weak force (blue) and strong force (red). Results from both USDL (solid lines) and SINDy [2] (dashed lines) are presented. Perfect reconstruction of the dynamical system is achieved when enough time-series are measured for both parameter regimes and both inference algorithms. However, SINDy algorithm requires less time-series in order to achieve perfect reconstruction in both regimes revealing that there are cases where iterative thresholding algorithm which is part of SINDy outperforms the greedy OMP algorithm which is part of the proposed approach. Moreover, the driving force F affects the performance of both algorithms for the same number of time-series. Higher value of F which implies more chaotic behavior results in higher inference accuracy. (b) RMSE as a function of the number of time-series for USDL (solid lines) and SINDy (dashed lines). Except when $F = 8$ and USDL algorithm (blue solid line), RMSE quickly reaches a plateau whose value quantifies the total discretization error. (c) ERC value for each state variable supplemented with confidence intervals. Weak (blue) and strong (red) forces are considered while one (dashed) or twenty time-series are feed to the algorithm. Greater values of ERC are observed under strong forcing revealing that chaos assists the correct inference of the dynamics. True recovery is theoretically guaranteed only for the strong forcing case and when at least 20 time-series are measured since then ERC is positive.

Figure 2(b) shows the RMSE between the true connectivity matrix and the estimated one for both algorithms and both parameter regimes. As expected, the RMSE decreases with the increase of the data size until it hits a plateau which stems from the discretization error of the various numerical integrations. Evidently, RMSE performance of USDL is better compared to SINDy at the strong force regime while the opposite is true at the weak force regime. Moreover, SSR metrics such as MIP and ERC correlate well with the performance of USDL algorithm as depicted in the subplots of Figure 2(c). ERC values per variable are (statistically) equal as expected because of the symmetry in the dynamical system's state variables. We remark also that theoretical guarantees on perfect reconstruction are satisfied only under strong forcing and at least $P = 20$ time-series of length 2 are taken into account since then

ERC is positive for all state variables. In contract, MIP is not small enough –it should be below 0.143– so as to theoretically guarantee perfect reconstruction.

Finally, we comment on the fine tuning of the hyperparameter for each approach. Each subplot of Figure 3 presents the F1 score (i.e., the harmonic mean of precision and recall) as a function of the hyperparameter value for both algorithms and forcing values. To avoid data leakage, the F1 score is estimated on a different set of data and not on the time-series used for the creation of Figure 2. We ubiquitously observe that the maximum F1 score increases as the number of time-series increases. Interestingly, the region of optimal hyperparameter values is wide showing the robustness of the sparse inference methods on the hyperparameter values.

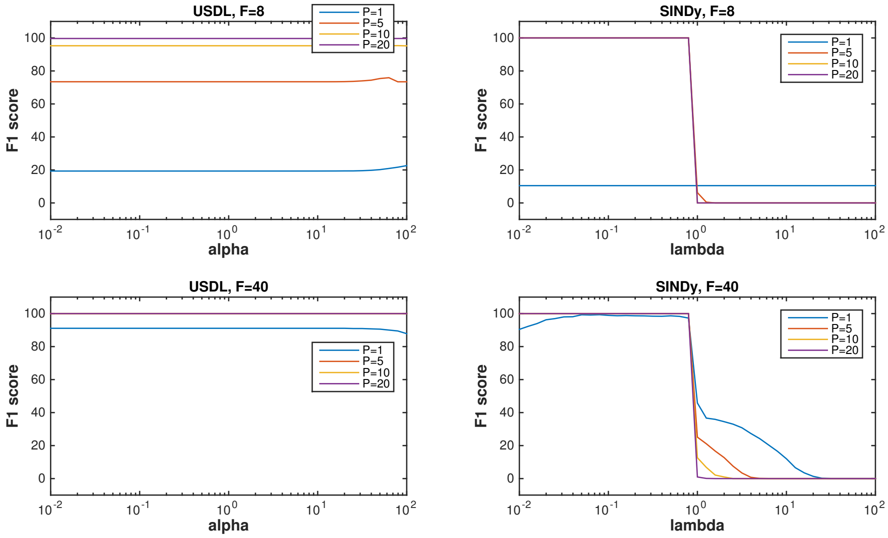

Figure 3: The harmonic mean between averaged precision and averaged recall (i.e., the F1 score) as a function of the hyperparameter for both USDL (left column) and SINDy (right column) and various number of time-series and forces. There is a large parametric region where optimal values are achieved for both algorithms.

Further Experimentation

We present a series of comparisons under various setups and assess the performance of the USDL algorithm. The first variation uses a different family of test functions keeping all the other hyperparameters fixed. A set of $M = 40$, equally-spaced basis spline (B-splines) functions of second-order are employed as test functions. Figure 4 presents the performance of the proposed approach in terms of precision and recall curves (upper left plot), in terms of RMSE (upper right plot) as well as metrics from SSR theory (lower plot). Results with B-splines are overall similar as in the case of Fourier modes. There is a minor difference with spline functions with USDL performing slightly better in terms of precision for weak forcing (blue in upper curve of Figure 4(a)) when the number of trajectories is in the range of 3-10. For comparison purposes, we also present the accuracy performance for SINDy algorithm which are the same as in Figure 2(a).

The second variation utilizes less test functions. Using less test functions is expected to weaken the strength of the inference method since less information is fed to the algorithm. Figure 5 shows the same quantities as in the first variation when only $M = 11$ Fourier modes are computed. Interestingly, the performance is similar to the case with 41 Fourier modes when at least $P = 5$ trajectories are provided for both weak and strong forces. The performance deteriorates significantly both in terms of precision and recall when the force is strong and one or two trajectories are measured. This behavior is in accordance with ERC which is significantly dropped from -0.8 to below -2 as lower panel of Figure 5(c) shows (dashed red line).

The third variation enriches the dictionary (i.e., $\psi(x)$) by considering additional candidate functions as driving forces of the dynamical system. Thus, apart from linear and quadratic terms, we add the complete set of cubic combinations resulting in $Q = 286$ dictionary atoms for the case of $N = 10$ state variables. Figure 6 presents the performance of both USDL (solid lines) and SINDy (dashed lines) algorithms upper plots) under this setup as well as the incoherence metrics (lower plots). Starting with the metrics of SSR theory, it is evident that MIP is

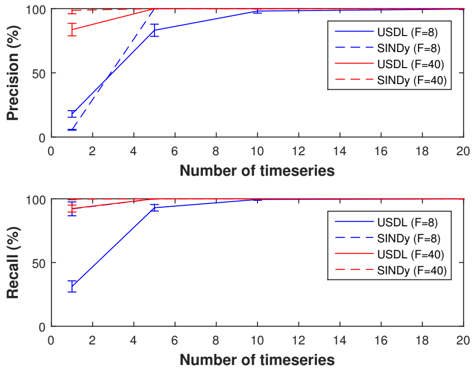

(a) Precision and recall

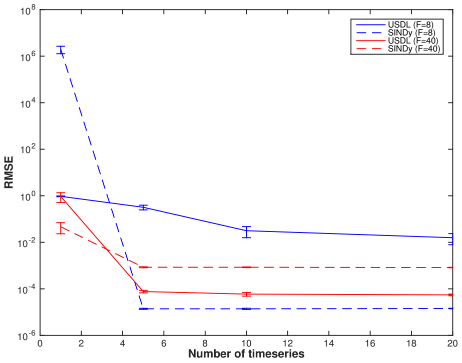

(b) RMSE

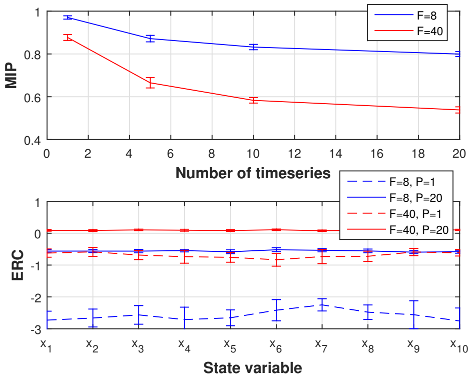

(c) MIP and ERC

Figure 4: Results for the Lorenz96 model when $M = 40$ and second-order basis spline functions are used as test functions. (a) Precision (upper panel) and recall (lower panel) curves as a function of P, i.e., the number of trajectories for both USDL (solid lines) and SINDy (dashed lines) algorithms. Performance of USDL algorithm is overall similar to the performance when Fourier modes are used. (b) RMSE as a function of the number of time-series for USDL (solid lines) and SINDy (dashed lines). It frequently occurs in our experiments that the RMSE for USDL algorithm when $F = 8$ (blue solid) is worse than when $F = 40$ (red solid) while the opposite frequently happens for the SINDy algorithm. (c) MIP (upper panel) and ERC per state variable (lower panel).

increased for both weak and strong forcing reflecting the fact that the additional atoms increase the collinearity of the measurement matrix, Ψ. In contrast, ERC remains almost the same. In terms of performance, both precision and recall deteriorate for the case of weak forcing (blue curves in Figure 6(a)). However, as the number of time-series is increased, the performance of both algorithms is greatly improved from almost 0 to almost 100 percent. For the strong forcing case (red curves in Figure 6(a)), apart from the case where very few trajectories are provided, perfect reconstruction of the dynamical system is observed showing once again that strong chaos assists the dynamical model inference. In contrast to the previous experiments, USDL algorithm performs better when the number of trajectories is low while SINDy algorithm performs better when the number of trajectories is above $P = 10$ where we observe perfect reconstruction of the dynamical system even for the weak force case (dashed blue lines in Figure 6(a)).

In all above variations, the RMSE performance were minimally affected by the different setups. The observed quantitative differences were in accordance with the precision-recall results. Moreover, we repeated the fine-tuning procedure for the hyperparameter values and the results were similar to Figure 3 therefore we keep the same values for both algorithms' hyperparameter.

The final comparisons demonstrate the effect of the sampling frequency on the sparse inference algorithms. Up to now, we considered a sampling rate of $1000Hz$ meaning that we sampled the time-series every 0.001 time units. Such a high sampling rate enabled us to assume that the time-series are accurate and merely continuous. In real applications though, high sampling frequency results in increased costs and typically there is a trade-off between expenses and sampling rate. Figure 7 presents various performance metrics as well as both SSR incoherence metrics

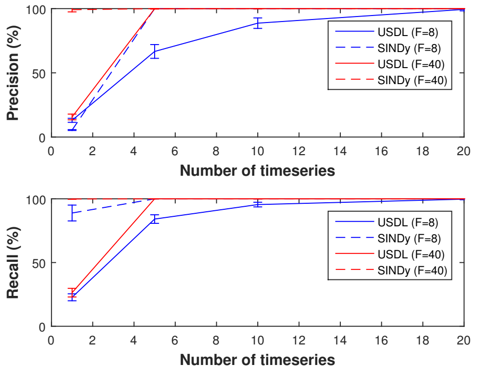

(a) Precision and recall

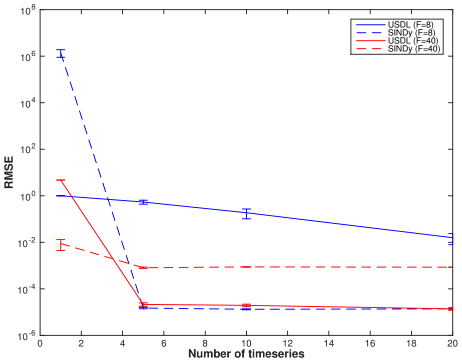

(b) RMSE

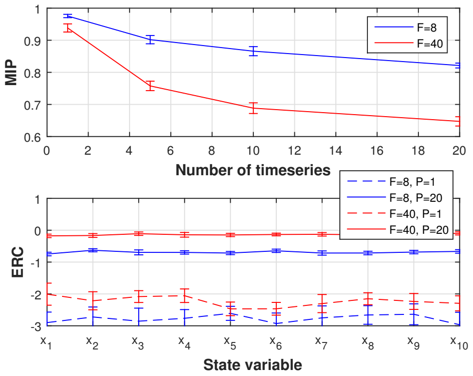

(c) MIP and ERC

Figure 5: Results for the Lorenz96 model when $M = 11$ Fourier modes are used as test functions. (a) Precision (upper panel) and recall (lower panel) curves as a function of P, i.e., the number of trajectories for both USDL (solid lines) and SINDy (dashed lines) algorithms. Even though four times less test functions are used, there is no or little deterioration of the performance in most of the cases ($P \geq 5$). (b) RMSE as a function of the number of time-series for USDL (solid lines) and SINDy (dashed lines). RMSE performance is similar to the previous experiment. (c) MIP (upper panel) and ERC per state variable (lower panel).

when sampling rate is dropped from $1000Hz$ to $100Hz$. Results are similar except for the strong forcing case with one trajectory where precision's performance decreases (red curves in upper panel of Figure 7(a)). The relative change of ERC (dashed red line in lower panel of Figure 7(c)) captures this deterioration. Notice that such deterioration is expected since strong forces result in larger and sharper modulations of the time-series. Again, SINDy algorithm achieves perfect reconstruction with less time-series compared to USDL algorithm. Overall, the performance is satisfactory when sampling frequency drops by a factor of 10. However, if the sampling frequency drops by a factor of 100, performance is severely reduced for USDL algorithm (solid lines) and moderately reduced for SINDy algorithm (dashed lines) as Figure 8(a) asserts. The increase of RMSE shown in Figure 8(b) further confirms the deterioration of the inference methods. There are two major factors contributing to this behavior. First, numerical integration produce larger error and, second, the time-series are sampled below the Nyquist frequency which resulted in aliasing and heavy distortion of the signals. Consequently, the dynamical system inference failed in this case revealing the sensitivity of the proposed method to adequate sampling. Finally, we would like to remark that we optimized over the hyper-parameter values using F1 score as a measure of goodness. The results on F1 score for both cases are shown in Figure 9 where it is also evidence the severe effects of over-downsampling (right plots). Moreover, the optimal parameter region has been shrunk implying that the inference methods are more sensitive to the hyperparameter value.

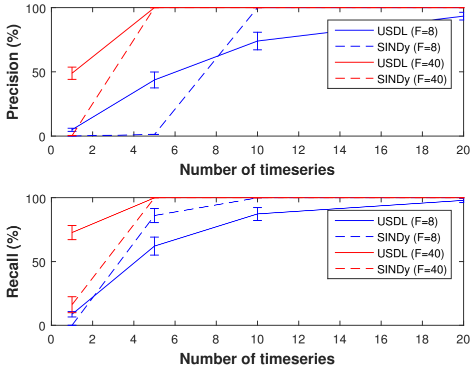

(a) Precision and recall

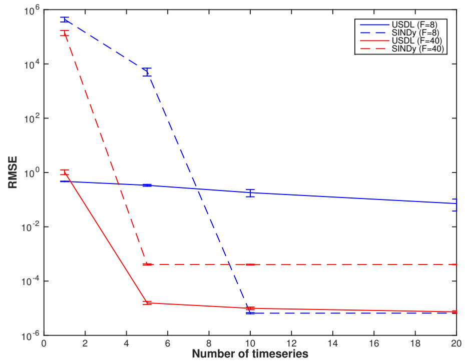

(b) RMSE

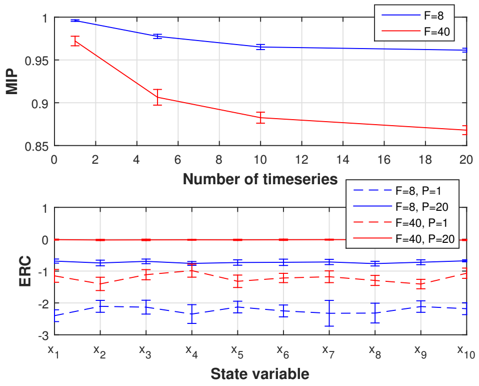

(c) MIP and ERC

Figure 6: Results for the Lorenz96 model when cubic terms are added to the dictionary. The size of the dictionary is approximately five times larger. (a) Precision (upper panel) and recall (lower panel) curves as a function of P, i.e., the number of trajectories for both USDL (solid lines) and SINDy (dashed lines) algorithms. Performance is slightly worse for the weak forcing case (blue) while perfect reconstruction is achieved when the force is strong (red) and more than $P = 5$ time-series are measured for both algorithms. Despite being less accurate for small P, SINDy algorithm is able to perfectly reconstruct the dynamical system under the weak force when $P = 10$ trajectories are provided. (b) RMSE as a function of the number of time-series for USDL (solid lines) and SINDy (dashed lines). RMSE results are in accordance with the precision and recall curves. (c) MIP (upper panel) and ERC per state variable (lower panel).

References

[1] Edward N. Lorenz. Predictability: a problem partly solved. In *Seminar on Predictability*, volume 1, pages 1–18, Shinfield Park, Reading, 1996. ECMWF.

[2] Steven L Brunton, Joshua L Proctor, and J Nathan Kutz. Discovering governing equations from data by sparse identification of nonlinear dynamical systems. *Proceedings of the National Academy of Sciences of the United States of America*, 113(15):3932–7, apr 2016.

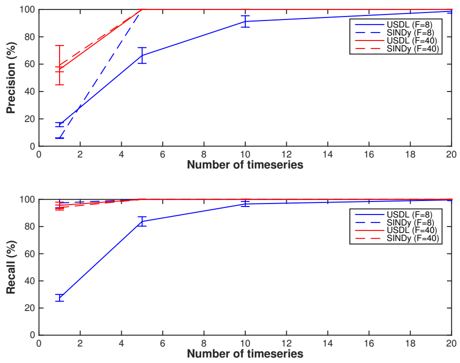

(a) Precision and recall

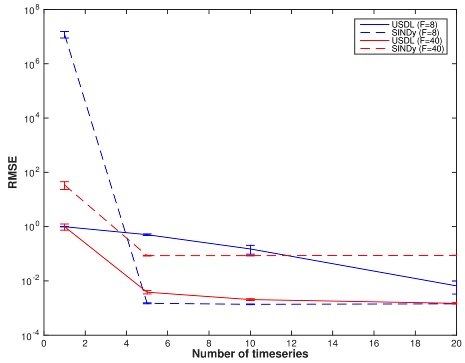

(b) RMSE

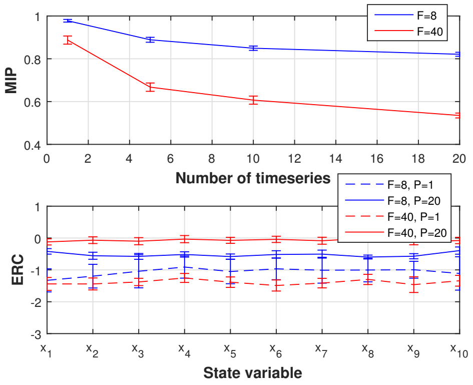

(c) MIP and ERC

Figure 7: Results for the Lorenz96 model when time-series are sampled at the rate of $100Hz$ instead of $1000Hz$. (a) Precision (upper panel) and recall (lower panel) curves as a function of P, i.e., the number of trajectories for both USDL (solid lines) and SINDy (dashed lines) algorithms. Minor reduction in the performance of both USDL and SINDy algorithms is observed. (b) RMSE as a function of the number of time-series for USDL (solid lines) and SINDy (dashed lines). Results are qualitatively similar to the case where the rate is $1000Hz$ (shown in Figure 2) while from a quantitative perspective RMSE is now two orders of magnitude worse compared to Figure 2. (c) MIP (upper panel) and ERC per state variable (lower panel) .

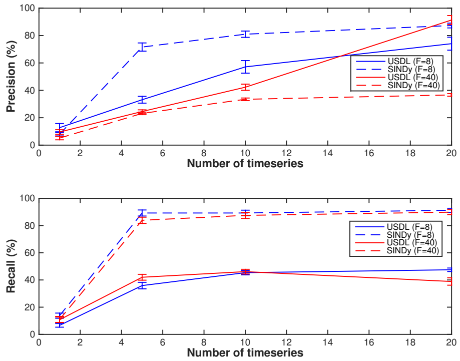

(a) Precision and recall

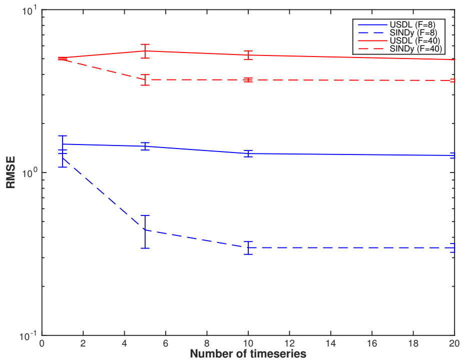

(b) RMSE

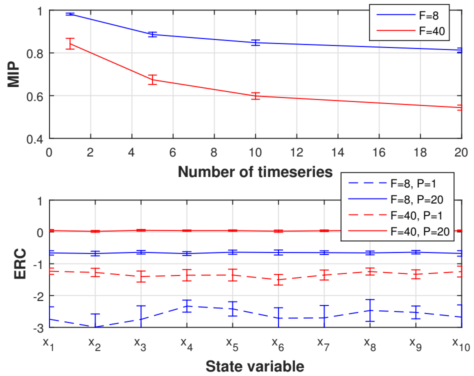

(c) MIP and ERC

Figure 8: Results for the Lorenz96 model when time-series are sampled at the rate of $10Hz$ instead of $1000Hz$. (a) Precision (upper panel) and recall (lower panel) curves as a function of P, i.e., the number of trajectories for both USDL (solid lines) and SINDy (dashed lines) algorithms. The impact of sub-sampling by a factor of 100 is huge, especially for the USDL algorithm, resulting in bad performance of the inference methods primarily in terms of precision. As expected, inference is more accurate under weak forcing due to insufficient sampling in the strong forcing case. (b) RMSE as a function of the number of time-series for USDL (solid lines) and SINDy (dashed lines). Stronger forces results in deterioration of RMSE performance for both algorithms. (c) MIP (upper panel) and ERC per state variable (lower panel).

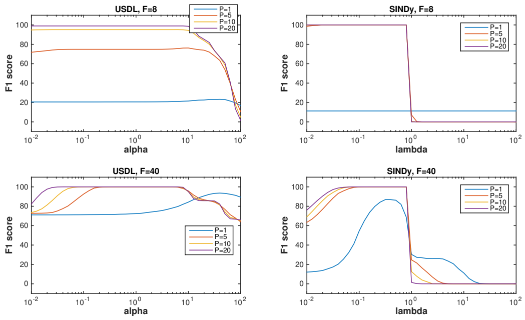

(a) F1 score with sampling frequency at $100Hz$.

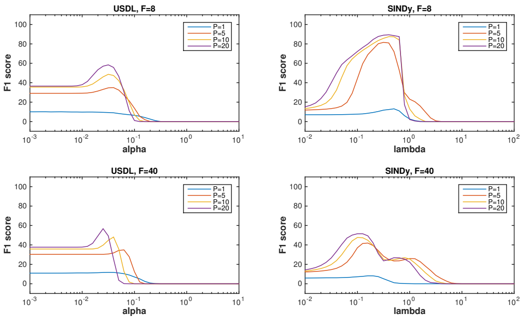

(b) F1 score with sampling frequency at $10Hz$.

Figure 9: (a) The F1 score as a function of the hyperparameter for both USDL (left column) and SINDy (right column) when the sampling rate of the time-series is at $100Hz$. The region of optimal values for the hyperparameter has been decreased. (b) Same as (a) with sampling frequency at $10Hz$. F1 score deteriorates significantly in all cases.
